# Dissecting RNA selectivity mediated by tandem RNA-binding domains

**DOI:** 10.1016/j.jbc.2025.108435

**Published:** 2025-03-20

**Authors:** Sarah E. Harris, Yue Hu, Kaitlin Bridges, Francisco F. Cavazos, Justin G. Martyr, Bryan B. Guzmán, Jernej Murn, Maria M. Aleman, Daniel Dominguez

**Affiliations:** 1 Department of Biochemistry and Biophysics, University of North Carolina, Chapel Hill, North Carolina, USA; 2 Department of Pharmacology, University of North Carolina, Chapel Hill, North Carolina, USA; 3 Department of Biochemistry, University of California, Riverside, California, USA; 4 Division of Biomedical Sciences, Center for RNA Biology and Medicine, Riverside, California, USA; 5 RNA Discovery Center, University of North Carolina, Chapel Hill, North Carolina, USA

**Keywords:** MSI1, MSI2, UNK, RRM, Zinc finger, RNA binding, competition, RNA bind-n-seq, Motif analysis

## Abstract

RNA–protein interactions are pivotal to proper gene regulation. Many RNA-binding proteins possess multiple RNA-binding domains; however, how these domains interplay to select and regulate RNA targets remains poorly understood. Here, we investigate three multidomain proteins, Musashi-1, Musashi-2, and unkempt, which share a high degree of RNA specificity, a common feature across RNA-binding proteins. We used massively parallel *in vitro* assays with unprecedented depth with random or naturally derived RNA sequences and find that individual domains within a protein can have differing affinities, specificities, and motif spacing preferences. We conducted large scale competition assays between these proteins and determined how individual protein specificities and affinities influence competitive binding. Integration of binding and regulation *in cells* with *in vitro* specificities showed that target selection involves a combination of the protein intrinsic specificities described here, but cellular context is critical to drive these proteins to motifs in specific transcript regions. Finally, evolutionarily conserved RNA regions displayed evidence of binding multiple RBPs *in cultured cells*, and these RNA regions represent the highest affinity targets. This work emphasizes the importance of *in vitro* and *in cultured cells* studies to fully profile RNA-binding proteins and highlights the complex modes of RNA–protein interactions and the contributing factors in target selection.

RNA-binding proteins (RBPs) are primary directors of post-transcriptional gene regulation where they bind and control RNA processing from synthesis, through maturation and degradation (1, 2, 3, 4). Over 1500 RBPs have been discovered to date within the human proteome (5). Much work has been done to catalog the RNA sequence preferences of these RBPs *in vitro* and identify targets *in cultured cells* (selected large-scale studies: Pantier *et al.* (6), Jolma *et al.* (7), Van Nostrand *et al.* (8), Dominguez *et al.* (9), Van Nostrand *et al.* (10), Ray *et al.* (11)). *In vitro* studies have largely found that RBPs tend to bind short (3–8 nucleotides) and relatively unstructured motifs within a given RNA. Additionally, determination of sequence preferences across RBPs have revealed limited motif diversity, with many RBPs binding similar motifs (9). However, *in cellulo*, short motifs are insufficient to fully explain target selection as they can be found in nearly every transcript (reviewed by Achsel and Bagni (12)). Instead, when a motif is present within the transcriptome, it is bound by a given RBP on average less than 10% of the time (9, 12, 13). Thus, RNA binding is not always explained by short sequence motifs (kmers or logos) but involves RNA structural preferences and sequence contextual features, including “split motifs,” that can be a challenge to identify and catalog from *in cellulo* experiments.

Further complicating RBP specificity is the fact that many RBPs are comprised of multiple RNA-binding domains (RBDs) in tandem, like RNA recognition motifs (RRMs), K homology (KH) domains, and zinc fingers (ZnFs) (5). When acting in concert, tandem RBDs can greatly enhance affinity and specificity towards RNA. In some cases, a single domain alone binds weakly (if at all) with μM affinity whereas two or more domains in tandem enhance binding strength, with some striking examples increasing affinity up to 10,000 fold (14, 15, 16, 17, 18, 19) (reviewed by Lunde *et al.* (20)). Cooperative binding by tandem domains has been reported for a number of RBPs (9, 21, 22, 23). For hnRNP A2/B1, the individual RRMs bind with low μM affinity, but the full protein achieves nM binding (22). Stitzinger *et al.* analyzed the cooperativity and avidity for seven multidomain RBPs including hnRNP A1 and IMP3 and found that tandem binding generally enhances affinity several fold (23). For PUM1, each of eight tandem repeats interacts with an individual nucleotide within the RNA target with high specificity and, in tandem, high affinity is achieved (14). A deep understanding of PUM1 binding has further enabled the generation of engineered PUMs with modified specificities, some of which contain more than eight repeats (14, 24, 25). However, much remains unknown when it comes to these tandem-domain RBPs: Are the affinities and specificities of individual RBDs within a protein similar? Does the ordering of RBDs within a protein impact binding? Can complex targeting *in cultured cells* be attributed to individual RBD-binding differences? Can dissection of individual RBDs and how they act together reveal more unique RBP-binding profiles than previously thought?

In the present study, we explore the RNA-binding preferences of three RBPs: Musashi-1 (MSI1), Musashi-2 (MSI2), and unkempt (UNK) (Fig. 1*A*). Each of these proteins have two tandem RBDs and their binding preferences have been previously investigated in several ways: individual crosslinking and immunoprecipitation (iCLIP) (26, 27, 28), RNA bind-n-seq (RBNS) (9, 21), x-ray crystallography (29), nuclear magnetic resonance (NMR) (30, 31), and fluorescence polarization (FP) (29, 32). All studies have found that MSI1, MSI2, and UNK tightly bind and regulate a core UAG motif. Despite their overlap in specificity, MSIs and UNK harbor different RBD types, two RRMs for MSI1 and MSI2 and six ZnFs for UNK (29, 30, 31, 33). All three RBPs also have important regulatory roles, where MSI1 and MSI2 control organ development, hematopoiesis, and stem cell maintenance (34, 35, 36, 37, 38, 39, 40, 41, 42, 43) and UNK modulates neuron morphology (26). Both MSI1 and MSI2 have been shown to drive cancer progression (44, 45) and UNK has further been implicated in neurological disease (26). All three proteins have been shown to regulate translation, as MSI1 and MSI2 can both positively and negatively influence mRNA target translation whereas UNK acts as a repressor (46, 47, 48).

**Figure 1:**
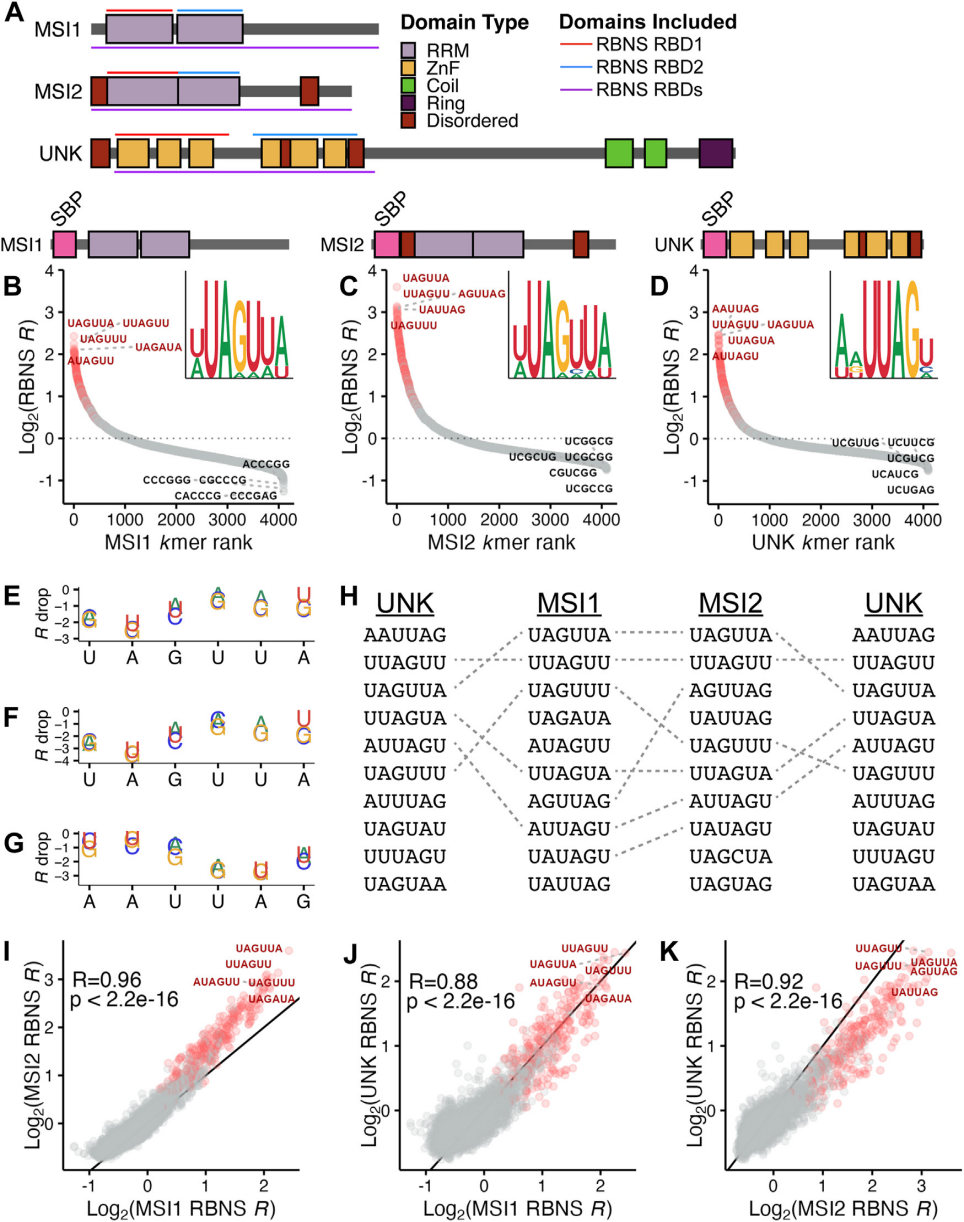
**Motif preferences for full-length MSI1, MSI2, and UNK *via* RBNS.***A*, domain architecture of MSI1, MSI2, and UNK as comprised of RRMs (*light purple*), ZnFs (*yellow*), coiled domains (*green*), ring domains (*dark purple*), and disordered regions (*dark red*). Regions used in full-length RBNS experiments are highlighted by *purple* lines, whereas regions used in individual domain RBNS experiments are highlighted by *red* and *blue lines*. *B*–*D*, scatter plot of all 6mer log_2_ enrichments for (*B*) MSI1, (*C*) MSI2, and (*D*) UNK as determined *via* RBNS. MSI1 and UNK assayed by Dominguez *et al.* (9). 6mers are colored by the presence of “UAG” (*red*) or none (*gray*). Logo was generated from the top 15 6mers. *E*–*G*, relative enrichment plot of each nucleotide within the top 6mer for (*E*) MSI1, (*F*) MSI2, and (*G*) UNK. *R* drop was calculated as the change in enrichment from the top 6mer upon mutation of a single nucleotide. *H*, overlap of the top 10 6mers for MSI1, MSI2, and UNK. *I*–*K*, correlation plots of (*I*) MSI1 *versus* MSI2, (*J*) MSI1 *versus* UNK, and (*K*) MSI2 *versus* UNK log_2_ enrichments. MSI1 and UNK data were obtained from GEO (9). 6mers are colored by the presence of “UAG” (*red*) or none (*gray*). Pearson's correlation coefficient and *p* value included.

Here, we examine these motif-overlapping yet functionally distinct proteins domain-by-domain to determine each domain's RNA-binding capabilities, a first step to understand how individual domains contribute to overall specificity, affinity, and independent target selection. As we dissect these proteins down to their tandem binding units, we find either retained motif specificity in the case of the RRMs of MSI1 and MSI2, or two different motifs for the ZnFs of UNK. Given the overlapping full-length specificity, we model and test how two of these proteins—MSI1 and UNK—compete for RNA targets using massive-scale biochemical assays. To contextualize binding patterns *in cellulo*, we reconstitute the interactomes of UNK and MSI1 *in vitro* and show that cellular binding patterns are not well-captured *in vitro*, suggesting that cellular factors are essential drivers of specific binding. Our studies highlight RBP functionalities that can arise from their individual RBDs and imply the complex cross-regulatory potential of RBPs with similar specificity in the cellular environment.

## Results

### UAG-binding proteins have similar specificities

To understand how individual RBDs contribute to affinity, we nominated three multidomain RBPs with similar RNA sequence specificity: MSI1, MSI2, and UNK (Fig. 1*A*). To test the similarity of binding preferences between these tandem domain containing proteins, we utilized RBNS, an unbiased massively parallel *in vitro* assay (9, 49). RBNS determines RNA sequence, secondary structure, and surrounding sequence context preferences of RBPs by allowing recombinant RBPs to select RNA from an extremely diverse pool of RNAs in a binding reaction (9, 49). RNA–protein complexes are subsequently isolated, bound RNA is sequenced, and sequence enrichments of protein-associated RNA are determined in relation to an input RNA pool (49). Additionally, RBNS does not rely on chemical or light-based crosslinking of protein to RNA such as CLIP-based methods and therefore is not impacted by crosslinking biases (reviewed by Chakrabarti *et al.* (50)). As these assays are performed *in vitro*, complex cellular factors are absent, and consequently, this method provides information on direct RBP–RNA interactions.

To confirm previous studies (9, 26, 27, 28, 29, 30, 31, 32), we obtained RBNS data from ENCODE for MSI1 and UNK (9) and individually assayed MSI2. We confirmed that all three proteins bind a UAG core motif with high specificity (Figs. 1, *B*–*D* and S1*A*). RBNS data provide a full spectrum of enrichment values (or *R* values, a proxy for affinity (49)) for all possible kmers, which is not easily achieved with crosslinking or possible with crystallographic methods. With the spectrum of enrichment values, further analysis of suboptimal kmers is possible. We calculated the relative importance of each position for the top 6mer by changing a base at each position and determining the consequence on enrichment (*R* drop). For all three proteins, disruption of the UAG had the greatest impact on binding whereas flanking sequences exhibited less severe penalties on *R* values (Fig. 1, *E*–*G*). The least severe change at the UAG was to either UAA or UAU, consistent with data to be presented below.

To determine the degree of shared specificity, we overlapped the top 10 6mers across these proteins and observed that at least 50% are shared between proteins (Fig. 1*H*). Of the top 25 bound 6mers, an average of 20 overlapped between two proteins and 15 overlapped across all three. The degree of overlapping specificity was also reflected by Pearson's correlation coefficients with all three sets of RBPs being highly correlated in kmer enrichments (MSI1 *versus* MSI2: R = 0.96, MSI1 *versus* UNK; R = 0.88, and MSI2 *versus* UNK: R = 0.92) (Fig. 1, *I*–*K*). These data emphasize the overlap of binding for these factors, including surrounding the UAG core motif.

### Individual domains of UAG-binding proteins contribute to protein individuality

Despite the top motifs for full-length MSI1, MSI2, and UNK having a UAG core, we hypothesized that the individual domains may have differing sequence preferences across these proteins to allow for protein-specific target selection as each protein has a distinct function in cells. To test this, we separated each protein into its individual RBDs—two RRMs for MSI1 and MSI2 and two ZnF clusters (ZnF1-3 and ZnF4-6) for UNK (33, 51)—and performed RBNS with these domains. We also utilized datasets we previously generated for UNK ZnF clusters (21) and replicated those RBNS assays for this study.

**Figure 2:**
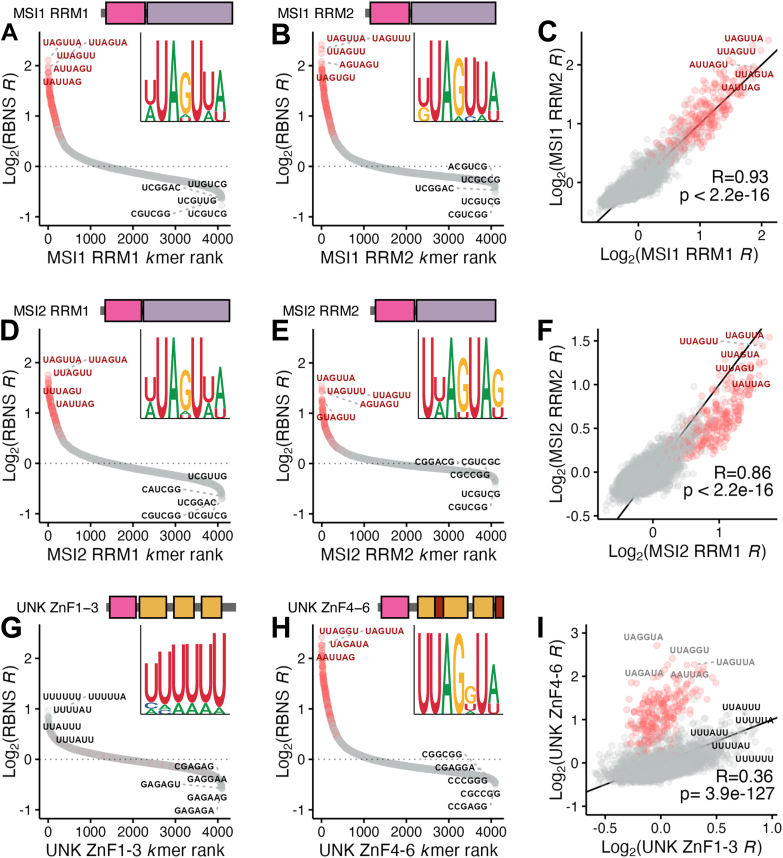
**Motif preferences for individual RBDs of MSI1, MSI2, and UNK *via* RBNS.***A* and *B*, scatter plot of all 6mer log_2_ enrichments for MSI1 (*A*) RRM1 and (*B*) RRM2 as determined *via* RBNS. 6mers are colored by the presence of “UAG” (*red*) or none (*gray*). Logo was generated from the top 15 6mers. *C*, correlation plot of MSI1 RRM1 *versus* MSI1 RRM2 log_2_ enrichments. 6mers are colored by the presence of “UAG” (*red*) or none (*gray*). Pearson's correlation coefficient and *p* value included. *D* and *E*, scatter plot of all 6mer log_2_ enrichments for MSI2 (*D*) RRM1 and (*E*) RRM2 as determined *via* RBNS. 6mers are colored by the presence of “UAG” (*red*) or none (*gray*). Logo was generated from the top 15 6mers. *F*, correlation plot of MSI2 RRM1 *versus* MSI2 RRM2 log_2_ enrichments. 6mers are colored by the presence of “UAG” (*red*) or none (*gray*). Pearson's correlation coefficient and *p* value included. *G* and *H*, scatter plot of all 6mer log_2_ enrichments for UNK (*G*) ZnF1-3 and (*H*) ZnF4-6 as determined *via* RBNS. Replicate data from this study was merged with that previously reported by Harris *et al.* (21). 6mers are colored by the presence of “UAG” (*red*) or none (*gray*). Logo was generated from the top 15 6mers. *I*, correlation plot of UNK ZnF1-3 *versus* UNK ZnF4-6 log_2_ enrichments. Replicate data from this study was merged with that previously reported by Harris *et al.* (21). 6mers are colored by the presence of “UAG” (*red*) or none (*gray*). Pearson's correlation coefficient and *p* value included.

From this, we found that both individual RRMs for MSI1 and MSI2 preferentially bind a UAG-core motif (Figs. 2, *A*, *B*, *D*, and *E* and S2, *A* and *B*), consistent with previous reports (9, 27, 28, 30, 31). The depth of our approach allowed analysis of suboptimal kmers as well. Of the top 25 6mers, 16 were cross-bound between MSI1's RRMs and 14 were cross-bound for MSI2's RRMs. kmer enrichments for MSI1's RRMs were more highly correlated than those of MSI2 (R = 0.93 and R = 0.86, respectively) (Fig. 2, *C* and *F*). We next sought to determine how amino acid conservation within domains could play a role in this domain-level RNA-binding specificity, as previous work has shown that 70% amino acid conservation can be predictive of binding specificity (11). However, while binding preferences for MSIs' RRMs are highly correlated, RRM1 and RRM2 conservation (percent identity) is only 45% for MSI1 and 42% for MSI2. To better understand this relatively low amino acid conservation with high binding similarity, we looked at an available NMR structure for MSI1 RRM2 in complex with RNA (30). We found that all RNA-contacting residues were conserved in identity between domains (52), save two which shared chemical similarity (as predicted by BLAST, D91 in RRM1 to E180 in RRM2, and R99 in RRM1 to K183 in RRM2; Fig. S2*D*). Therefore, the tandem domains of MSI1 and MSI2 bind the same core motif not through amino acid sequence conservation but by conservation of protein structure and RNA contacts.

For UNK, we observed that ZnF1-3 preferentially binds an A/U rich motif whereas ZnF4-6 associates with the primary UAG core (Figs. 2, *G* and *H* and S2*C*). We and others have previously observed these patterns (9, 21, 26, 29). Due to these separate preferences between the individual domains, their binding patterns are not highly correlated (Fig. 2*I*). Of the top 25 bound 6mers for each domain, none of them are cross-bound between ZnF1-3 and ZnF4-6, and further the enrichment values for ZnF1-3 were overall lower, suggesting a lower affinity for RNA by ZnF1-3. When aligning ZnF1-3 and ZnF4-6 domains with BLAST (52), little amino acid conservation exists across domains; however, the key RNA-contacting residues as defined with crystal structures (29) are topologically in the same position despite a lack of sequence conservation (Fig. S2*E*). This conservation contrasts with the MSI1 RRMs and could explain the lack of retained RNA specificity and affinity across UNK's RNA-binding units. These data support a complex model wherein tandem RBDs can have overlapping (MSIs) or distinct (UNK) motif specificities, while specificities of the full-length proteins are largely similar.

### Spacing and contextual preferences vary for UAG-binding proteins

While we have shown the top motif for each RBD unit and full-length protein, there are many short 3mers that are well-bound by each RBD independently (Fig. S2, *A*–*C*). The affinity of each RBD to these sequences, the spacing of these motifs, and the structural context in which they are embedded are all expected to generate a large repertoire of RNA targets with similar affinities. Therefore, we sought to investigate the motif spacing preferences for full-length MSI1, MSI2, and UNK in an unbiased high-throughput manner.

**Figure 3:**
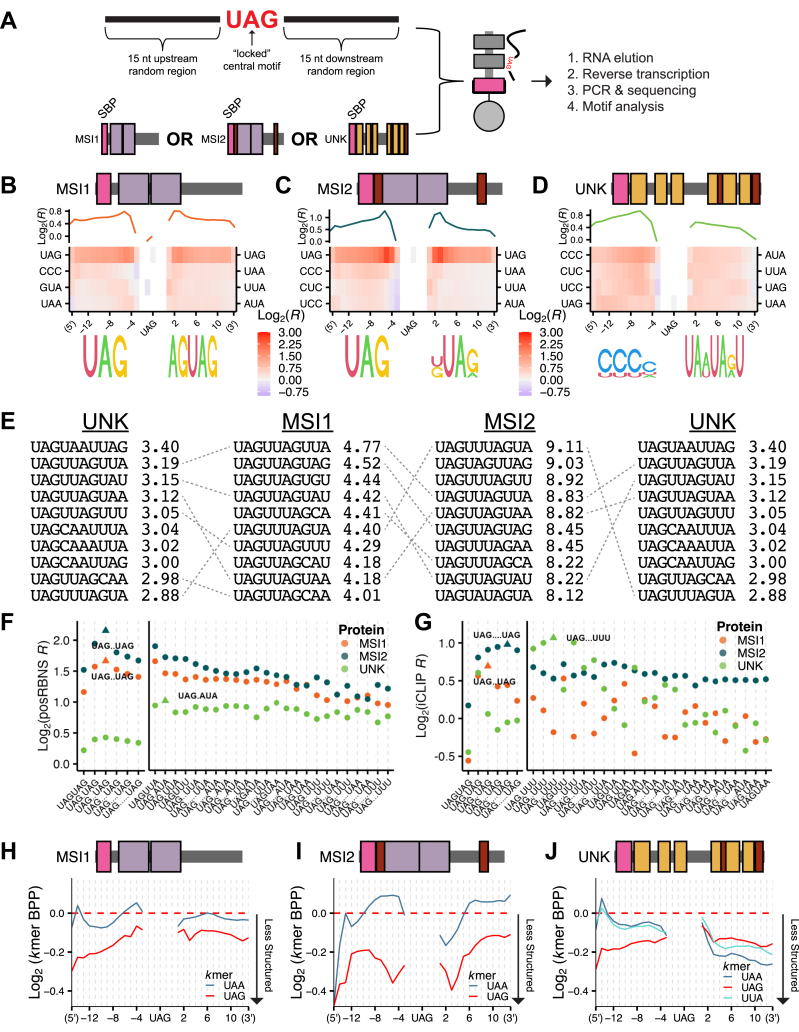
**Secondary motif, spacing, and base pair probability preferences for MSI1, MSI2, and UNK *via* posRBNS.***A*, design of RNA pool and layout of posRBNS. *B*–*D*, heat map of the log_2_ posRBNS enrichment of top four enriched 3mers for (*B*) MSI1, (*C*) MSI2, and (*D*) UNK. Logos were generated from the top five 4mers. Log_2_(*R*) line plot shows average enrichment of top four 3mers positionally. *E*, overlap and enrichment of the top ten UAGN_7_ 10mers for MSI1, MSI2, and UNK. posRBNS enrichment values shown. *F*, Log_2_ posRBNS enrichment of bipartite motifs for MSI1 (*orange*), MSI2 (*blue*), and UNK (*green*). Top bipartite motif and spacing for each protein are denoted by *triangles*. *G*, Log_2_ iCLIP enrichment of bipartite motifs for MSI1 (*orange*), MSI2 (*blue*), and UNK (*green*). Top bipartite motif and spacing denoted by *triangle*. *H*–*J*, line plot of the base pair probability of the top two enriched 3mers for (*H*) MSI1, (*I*) MSI2, and (*J*) UNK.

To address this question, we developed a version of RBNS (positional RBNS or posRBNS) where the preferred motif is locked centrally, and the surrounding nucleotides are randomized during DNA synthesis (Experimental procedures; Fig. 3*A*), resulting in very diverse pools of RNAs each with a central UAG. This approach increases our ability to determine complex motifs, motif spacing, and RNA secondary structure by enabling the analysis of millions of bound sequences all containing the UAG of interest, making it a more ideal assay for multidomain proteins. For full-length MSI1 and MSI2, as expected given our results above, the top secondary motif outside of the core-UAG motif is an additional UAG motif (Figs. 3, *B* and *C* and S3, *A* and *C*). A spacing preference of two nucleotides between UAGs was observed, but positive enrichments were still observed with much larger spacings, indicative of the flexibility MSIs have in target selection. To determine how other motifs may influence the interaction, we filtered our data such that only the central UAG was present (*i.e.*, no reads with another UAG anywhere else in the sequence were included) and asked what secondary motifs were enriched. UAA, UUA, and AUA were enriched downstream while GUA, UAA, and CCC were enriched upstream for the MSI paralogs. For UNK, we observed a downstream A/U-rich preference with variable spacing between one and four nucleotides (Figs. 3*D* and S3*E*). Upstream of the locked UAG core, we conversely observed a strong preference for C-rich motifs, again with variable spacing. We have little evidence that these C-rich motifs are directly bound by these RBPs but instead believe them to be modifiers of RNA secondary structure as discussed further below.

Given the depth of this experiment, we were able to analyze 10mers starting with the locked UAG (*e.g.* UAGN_7_). This type of analysis has previously been a challenge given that there are 4^10^ (∼1e6) possible 10mers and quantifying the full spectrum of 10mer enrichments with robustness is a significant challenge due to most experiments lacking necessary depth. However, since we are most interested in UAG-containing 10mers and every sequence has a UAG, posRBNS is well suited for this analysis. Across all three proteins, 10mer analysis demonstrated the highest enrichments for UAGN_1-2_UAG where N was preferentially U/A (see figure for specific sequences; Fig. 3*E*). Further, all three proteins exhibit strong binding patterns *in vitro* to UAGN_1-5_UAG and UAGN_1-5_(A/U-rich) (Fig. 3*F*). Strikingly, even though there are a total of 16,000 UAG-containing 10mers, the top 10mers were still largely shared for each full-length RBP (Fig. 3*E*).

To test how these *in vitro* patterns compared to patterns *in cellulo*, we examined available iCLIP data for all three proteins (26, 27, 28). We identified iCLIP peaks for each protein, determined their transcript region, and selected “control peaks” matched for transcript region. Similar to the RBNS approach, we next computed motif enrichments within protein-bound peaks relative to control peaks (Experimental procedures). We observed similar enrichment patterns between posRBNS and iCLIP where MSI1 and MSI2 preferentially bound to bipartite UAG motifs (with varied spacing), and UNK preferentially bound to UAGN_3_UUU (Fig. 3*G*). While these patterns are slightly different from what was observed strictly *in vitro*, *in vitro* patterns for each protein are still highly frequent *in cellulo*. Further, we cannot exclude that crosslinking biases that generally favor U's may impact the detection of motifs in iCLIP.

To understand if any RNA structural preferences impact binding patterns, we performed *in silico* RNA folding (RNAfold (53)) and determined the base pair probability (BPP, likelihood that each base is paired within a given RNA) of every 3mer in our posRBNS experiment. Top motifs (UAG and UAA for MSI1 and MSI2; UAA, UAG, and UUA for UNK) displayed lower BPP (less structured, more accessible) downstream of the locked central UAG motif (Figs. 3, *H*–*J* and S3, *B*, *D*, and *F*), likely contributing to increased binding due to the unstructured RNA preferences for many RBPs (7, 9). As noted above, we observed C-rich motifs as upstream enriched for all three proteins. We hypothesized that this abundance may be due to the intrinsic nature of C to not pair with downstream A/U-rich regions, thus promoting motif accessibility. We found that CCC motifs largely have the lowest BPP of all kmers upstream of the central UAG (Fig. S3, *G*–*I*), supporting the hypothesis. Taken altogether, MSIs and UNK display specific (UAG and U/A-rich requirements) but flexible (variable spacing) binding that is likely mediated through the action of these tandem RBDs.

### MSI1, MSI2, and UNK demonstrate similar binding affinities and multidomain avidity

**Figure 4:**
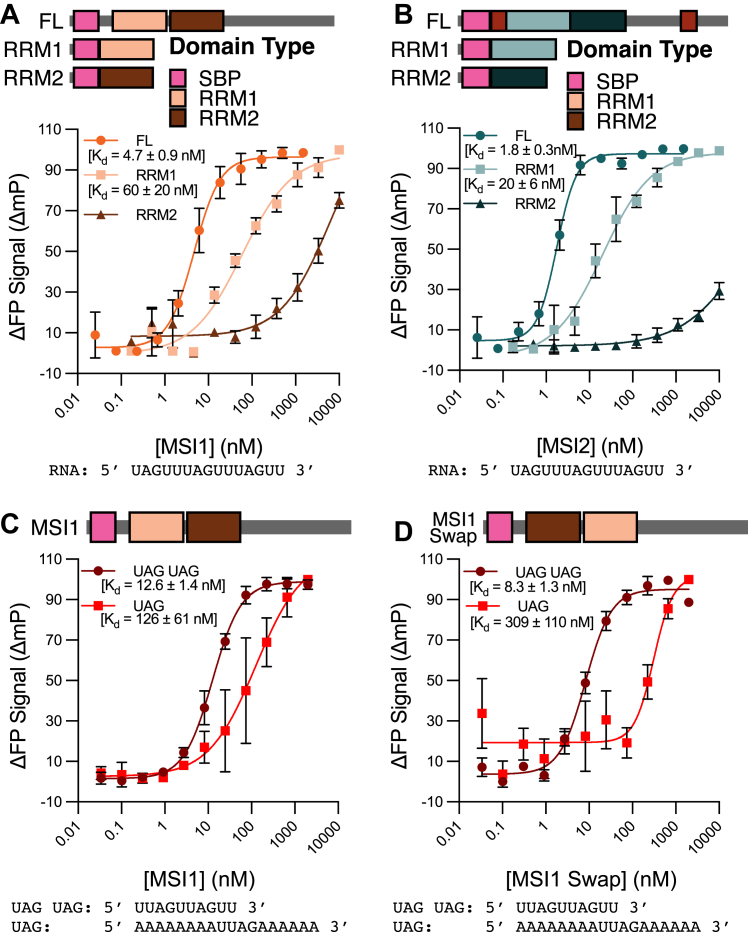
**Affinity of MSI1 and MSI2 for UAG-containing sequences.***A*, delta fluorescence polarization–binding curves (n = 3) for full-length MSI1 (*orange circle*), MSI1 RRM1 (*light orange square*), and MSI1 RRM2 (*dark orange triangle*) incubated with a tri-UAG–containing oligo. Full-length curve was normalized to its minimum and maximum fluorescence polarization signal with RRM1 and RRM2 normalized to the maximum of RRM1 to produce delta fluorescence polarization values. Data are presented as mean values ± SD. *B*, delta fluorescence polarization–binding curves (n = 3) for full-length MSI2 (*teal circle*), MSI2 RRM1 (*light teal square*), and MSI2 RRM2 (*dark teal triangle*) incubated with a tri-UAG–containing oligo. Full-length curve was normalized to its minimum and maximum fluorescence polarization signal with RRM1 and RRM2 normalized to the maximum of RRM1 to produce delta fluorescence polarization values. Data are presented as mean values ± SD. *C* and *D*, delta fluorescence polarization–binding curves (n = 3) for full-length (*C*) MSI1 and (*D*) MSI1 swap incubated with a mono-UAG–containing oligo (*red square*) or a dual-UAG–containing oligo (*dark red circle*). Each curve was normalized to its minimum and maximum fluorescence polarization signal to produce delta fluorescence polarization values. Data are presented as mean values ± SD.

To evaluate affinities across full-length proteins and RNA-binding domains, we used FP between recombinant proteins and UAG-containing RNA oligos. We previously showed that UNK binds this RNA sequence at 40 nM (21). When performing FP with MSI1 or MSI2, we observe higher affinity for this sequence as MSI1 binds at 5 nM and MSI2 at 2 nM (Fig. 4, *A* and *B*). Interestingly, this affinity difference between MSI1 and MSI2 was also observable *via* RBNS as MSI2 exhibited higher average enrichments than MSI1 (Fig. S1*A*). To test how each domain contributes to affinity, we assessed domains individually. When doing this previously with UNK, we observed that ZnF4-6 bound a UAG-containing oligo approximately 5-fold better than ZnF1-3 (0.4 *versus* 2.4 μM, respectively (21)). Interestingly, we observed similar patterns for MSI1 and MSI2 where one RRM bound with greater affinity (more than 5-fold better) than the other (Fig. 4, *A* and *B*). These data present a potential “tandem anchoring” mechanism applicable for other multidomain RBPs where one domain mediates primary strong contacts (large anchor) with high affinity for a given RNA sequence and the second domain serves to enhance avidity to the RNA target (stabilizing anchor). Indeed, previous studies have highlighted similar cooperative potential between RBDs (17, 54, 55, 56, 57, 58) (reviewed by Lunde *et al.* (20) and Auweter *et al.* (59)). We additionally tested binding of full-length and RBDs of MSI1, MSI2, and UNK against random sequence RNA (N_16_) to determine if the RNA backbone contributed significantly to binding. While all three full-length proteins demonstrated some binding potential at high protein concentrations, these binding affinities were drastically lower than the affinities for UAG-containing RNAs (Fig. S4, *A*–*C*).

We further tested binding of MSI1 with varying combinations of UAG motifs. With a mono-UAG–containing oligo, we fit a K_d_ of 126 nM. However, the binding is enhanced 10-fold with the addition of one more UAG, likely driven by the dual-RRM architecture of MSI1 (Fig. 4*C*). As both RRMs of MSI1 preferentially bind a UAG-core motif, we next asked whether orientation of these domains was important for MSI1 binding. We observe that by swapping the order of the domains, the affinity for a mono-UAG–containing oligo is decreased 2-fold. However, if the RNA can accommodate both domains (*i.e.*, possesses two UAG motifs), no observable K_d_ difference is present upon this domain swap (Fig. 4*D*).

### Bipartite RNA motifs allow for increased affinity for multidomain RNA-binding proteins

**Figure 5:**
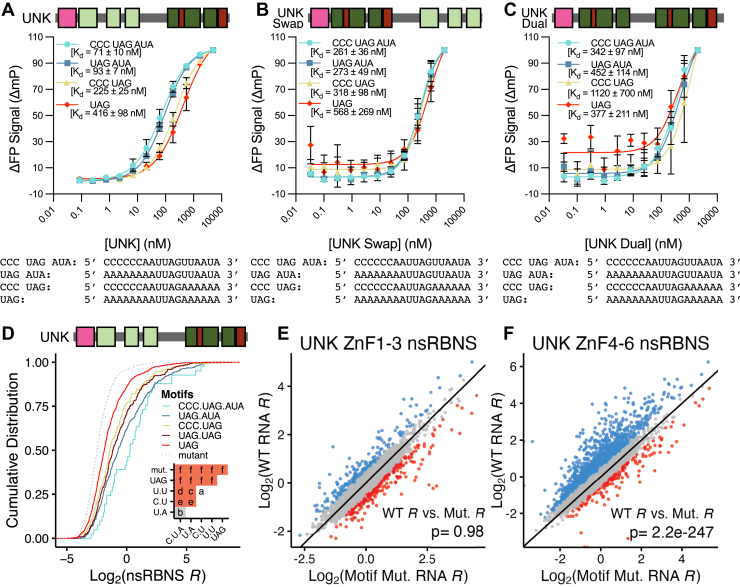
**Combinatorial affinity of UNK for positional motifs.***A*–*C*, delta fluorescence polarization–binding curves (n = 3) for (*A*) UNK, (*B*) UNK swap, and (*C*) UNK dual incubated with a UAG-containing oligo (*red diamond*), CCC-UAG–containing oligo (*yellow triangle*), UAG-AUA–containing oligo (*blue square*), or a CCC-UAG-AUA–containing oligo (*cyan circle*). Each curve was normalized to its minimum and maximum fluorescence polarization signal to produce delta fluorescence polarization values. Data are presented as mean values ± SD. *D*, cumulative distribution function of log_2_ nsRBNS enrichments with UNK of positional motif–containing oligos from Harris *et al.* (21): CCC-UAG-AUA (*cyan*), UAG-AUA (*blue*), CCC-UAG (*yellow*), UAG-UAG (*dark red*), UAG (*red*), motif mutant (*light gray, dotted*). Inset shows significance values for all comparisons *via* two-sided KS test and corrected for multiple comparisons *via* the BH procedure. *Red* denotes significant (*p* ≤ 0.05), and *gray* denotes nearing significant (*p* ≤ 0.1). Values are as follows: a (ns), *B* (*p* ≤ 0.1), *C* (*p* ≤ 0.05), *D* (*p* ≤ 0.01), *E* (*p* ≤ 0.001), *F* (*p* ≤ 0.0001). *E*–*F*, scatter plot of log_2_ nsRBNS enrichments with (*E*) UNK ZnF1-3 and (*F*) UNK ZnF4-6 of WT (Y-axis) *versus* motif mutant (X-axis) oligos. Log_2_ change in enrichment (wt-mut) was calculated for each sequence pair: >0.5 defined as bound better in wt (*blue*), <−0.5 defined as bound better in mut (*red*), 0 ± 0.5 defined as similar binding (*gray*). Significance determined *via* paired, one-sided Wilcoxon test.

As previously shown, UNK is distinct in this study from MSI1 and MSI2 in that its “two” RBDs prefer separate sequences: ZnF4-6 binding a UAG-containing sequence, and ZnF1-3 binding more general A/U-rich RNA. Therefore, we designed RNA oligos based on the findings from the posRBNS (Fig. 3*D*) to test the dual-domain avidity for UNK. UNK weakly binds to a mono-UAG oligo with an affinity of 400 nM; however, adding in a downstream AUA enhanced binding affinity to 90 nM. As noted above, the posRBNS revealed the enrichment of Cs upstream of the UAG, which were subsequently added and enhanced UNK affinity by 2-fold. However, upstream Cs had little impact on binding affinity on oligos that already contained both the core UAG and downstream AUA. (Fig. 5*A*). Despite these sequences being able to enhance binding, neither polyC nor polyU oligos demonstrated any binding on their own (Fig. S5*A*), suggesting that UNK is binding the core UAG, and the interaction is then modestly enhanced by these upstream Cs and substantially enhanced by downstream AU-rich sequences as suggested by the posRBNS *in vitro*.

To assess how domain order contributes to binding and further confirm each domain's preferential sequences, we tested the same RNA oligos against both a swapped order domain construct of UNK (UNK Swap, ZnF4-6, 1–3) and an UNK construct with two ZnF4-6 domains (UNK Dual, tandem Znf4-6) (Fig. 5, *B* and *C*). However, the swapped order construct and dual construct failed to recapitulate the full-strength binding of the UNK, falling short of the highest affinity RNA by >3-fold. In contrast to the MSI1, these data highlight the importance of domain order to RNA binding for UNK RBDs and likely other RBPs with less similar tandem RBDs.

We previously designed a natural sequence version of RBNS (nsRBNS) based on iCLIP data (26) to test how UNK-binding patterns change between human and mouse (21). We revisited these data to determine the enrichment of natural targets containing the above-described motifs. Strong enrichments of UNK to CCC-UAG-AUA and UAG-AUA-containing sequences over a single UAG or two UAGs in tandem were observed (Fig. 5*D*), consistent with our posRBNS findings. As part of this nsRBNS design, sequences wherein the UAG was mutated to CCG were included (mutant), and as shown (Fig. 5*D*), mutation of UAGs abolished binding, supporting that despite the presence of secondary motifs, a UAG is still required for the interaction.

To further highlight the differences between ZnF1-3, ZnF4-6, and full-length UNK on binding against natural sequences, we performed nsRBNS using an UNK-specific RNA pool similar to the one described above (Experimental procedures) (21). We rationalized that assaying against the individual domains would allow for an understanding of how each domain contributes to the global binding patterns observed for UNK *in cellulo*. We calculated the Pearson's correlation coefficient for each domain *versus* full-length UNK in our nsRBNS assay (21). We observe weak correlation for ZnF1-3 *versus* full-length (R = 0.27) and moderate correlation with ZnF4-6 *versus* full-length (R = 0.56) (Fig. S5, *B* and *C*). Generally, ZnF1-3 displayed overall weaker enrichments than ZnF4-6 (Fig. S5*D*), consistent with the affinity differences between these domains observed by FP. Using the mutant sequences within the nsRBNS library, we hypothesized that ZnF1-3 would remain agnostic to UAG motif mutants (since it binds AU-rich sequences), whereas ZnF4-6 would be largely impacted. Indeed, no large binding differences were observable for ZnF1-3 (Fig. 5*E*), whereas ZnF4-6 largely displayed preferential binding to the WT oligos (Fig. 5*F*). These data demonstrate that while neither domain could recapitulate the full binding strength of the whole protein, ZnF4-6 is more similar to full-length UNK and therefore a stronger mediator of binding natural UNK-bound sequences. These data provide further evidence towards the “tandem-anchoring” hypothesis. Using UNK as a model, ZnF4-6 targets UNK to the desired RNA motif, and ZnF1-3 provides secondary anchoring and stabilization for the interaction.

### MSI1 and UNK compete for some binding sites *in vitro*

**Figure 6:**
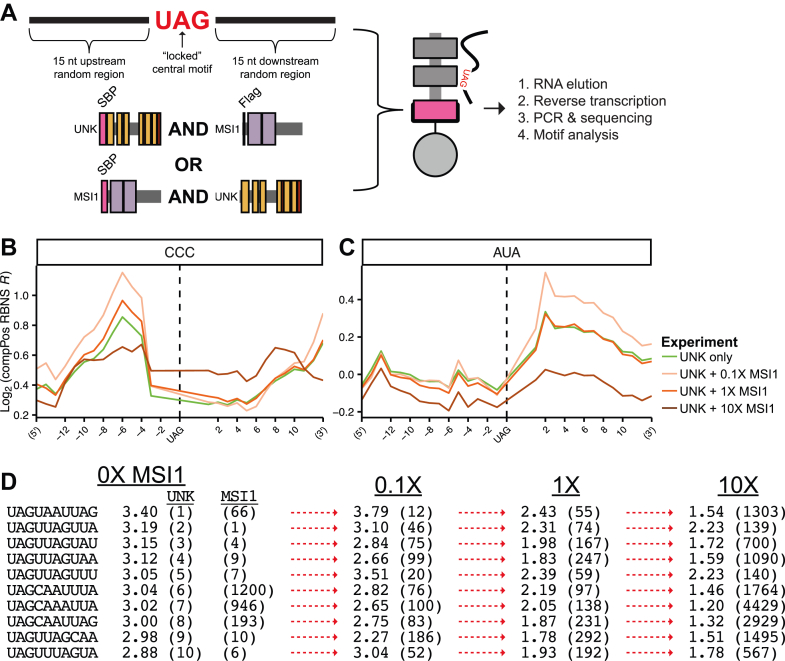
**Competition of secondary motifs between MSI1 and UNK as determined *via* Competition posRBNS.***A*, design of RNA pool and layout of competition posRBNS. *B* and *C*, Log_2_ enrichments of oligos containing (*B*) CCC and (*C*) AUA bound to UNK with MSI1 in competition at 0 (*green*), 0.1× (*light orange*), 1× (*orange*), or 10× (*dark orange*). *D*, rank depletion of the top ten UAGN_7_ 10mers for UNK with MSI1 in competition. *Arrows* denote changes to rank with *red* denoting loss of top 10 rank (new rank shown in parentheses next to enrichments).

Thus far, we have highlighted similarities and differences between MSI1, MSI2, and UNK and find that they can bind nearly identical motifs with high affinity (Figs. 1 and 4). This raises the possibility that at times, these proteins may compete for targets. Further, both MSI1 and UNK are expressed in the same cell types (60), can localize in the cytoplasm, and are both involved in translational regulation. To determine how these proteins bind RNA in competition, we designed competition posRBNS (compPosRBNS) similar to posRBNS as described above, but in the presence of increasing competitor protein. This competitor protein (UNK for MSI1-centric or *vice versa*) was produced without an SBP tag, so only the assayed protein and bound RNAs would be captured with magnetic pulldown while the competitor titrates away RNA targets (Fig. 6*A* and Experimental procedures). This method allows us to simulate a more complex, cell-like environment where multiple proteins and multiple targets are present, allowing for more precise and optimal target selection. Starting with SBP-UNK, we titrated in 0, 0.1, 1, or 10× MSI1 and measured the effects of RNA binding to UNK (Fig. 6, *B*–*D*). In this experiment, we observe that UNK-specific sequences—like upstream CCC (Fig. 6*B*) or downstream AUA (Fig. 6*C*)—are difficult to compete off with MSI1 and only see depletion at 10× competitor. We noted some apparent enhancement of UNK binding to CCC or AUA at lower MSI1 concentrations (discussed in more detail below). Further, when considering only oligos with a single UAG, competition patterns are still observable, indicating that MSI1 can compete for UNK targets *in vitro* even when MSI1's sequence preferences (*i.e.*, dual-UAG) are not met (Fig. S6, *A* and *B*).

Similar to the posRBNS analysis above, we calculated enrichment values for all 10mers starting with UAG (UAGN_7_). We compared both 10mer enrichments and ranks (*e.g.*, 1 being the top 10mer and so forth) with varying competitor concentrations. All of UNK's top 10mers readily change in rank even at 0.1× MSI1, and significant drops in enrichments are seen at higher protein concentrations (Fig. 6*D*). UNK's top preferred 10mer (UAGUAAUUAG) is not as readily competed off (rank of 1–12), indicative of UNK maintaining some binding to the preferred motif even in the presence of MSI1. However, other 10mers drop hundreds of positions at low MSI1 concentrations and over a thousand positions at the highest MSI1 concentrations. Interestingly, we found that at the low MSI1 concentration 0.1×, UNK enrichments actually increased (while ranks drop). We believe this effect to be related to low levels of competitors titrating away suboptimal binders, thereby enhancing the enrichment of higher binding sequences similar to improving signal-to-noise.

When performing this assay in the opposite direction with SBP-tagged MSI1 and untagged UNK as competitor, we see that competition is not readily apparent (Fig. S6, *C*–*F*). This is likely driven by MSI1's greater affinity for RNA than that of UNK's (Fig. 4*A*). Very minor changes are observed for UNK's secondary motifs (CCC and AUA; Fig. S6, *C* and *E*), whereas UNK is unable to compete with MSI1 for bipartite UAG motifs (Fig. S6*D*). When comparing 10mer rank across competition concentrations, very few large rank changes are observed (Fig. S6*F*). When taken together, compPosRBNS data highlight how sequence selectivity and affinity of RBPs play into competitive binding aspects *in vitro*, providing potential biological implications for their cross regulation within cells.

### MSI1 and UNK demonstrate some overlapping transcript specificity in cultured cells

Understanding and modeling the biochemical details of how proteins bind RNA may provide a better understanding of how targets are selected *in cellulo*. However, to achieve this, natural sequences with evidence of binding in cells need to be assessed *in vitro*. We previously investigated species-specific UNK-RNA–binding patterns between human and mouse using a massive scale *in vitro* assay involving thousands of natural RNA targets derived from iCLIP (21). We sought to apply this approach to examine how RBPs that target similar *in vitro* sequences (*i.e.*, MSI1 and UNK) select for their targets *in cellulo*.

**Figure 7:**
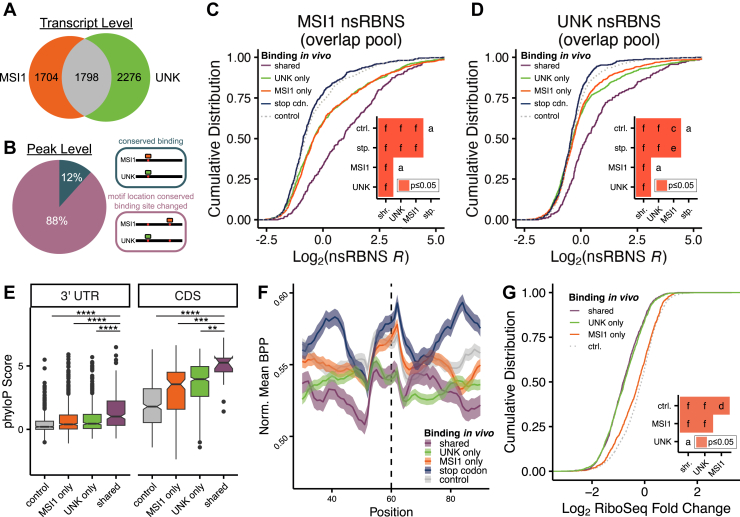
**Analysis of MSI1 and UNK competition potential *in cellulo* and *in vitro*.***A*, transcript level conservation of iCLIP hits between MSI1 (from glioblastoma) (27) and UNK (from SH-SY5Y or UNK-overexpressing HeLa) (26). *B*, motif level conservation of iCLIP hits between MSI1 (from glioblastoma) (27) and UNK (from SH-SY5Y or UNK-overexpressing HeLa) (26). *C* and *D*, cumulative distribution function of log_2_ nsRBNS enrichments with (*C*) MSI1 or (*D*) UNK of all iCLIP hits: control (*light gray*; *dotted*), stop codon (*dark blue*), MSI1 only (*orange*), UNK only (*green*), and shared (*purple*) oligos. Insets show significance values for all comparisons *via* two-sided KS test and corrected for multiple comparisons *via* the BH procedure. *Red* denotes significant (*p* ≤ 0.05). Values are as follows: *A* (ns), *C* (*p* ≤ 0.05), *E* (*p* ≤ 0.001), *F* (*p* ≤ 0.0001). *E*, box and whisker plot of the phyloP score of nsRBNS oligos: control (*light gray*), MSI1 only (*orange*), UNK only (*green*), and shared (*purple*). *Center line* denotes median (50th percentile) with bounds of box representing 25th to 75th percentiles and the whiskers denoting 5th to 95th percentiles. Outliers are denoted as individual points. *Asterisks* denote significance as determined *via* two-sided Wilcoxon test and are as follows: ∗∗ (*p* ≤ 0.01), ∗∗∗ (*p* ≤ 0.001), ∗∗∗∗ (*p* ≤ 0.0001). *F*, Log_2_ fold change of mean base pair probability with a 10-nucleotide sliding window of the central region of shared, UNK only, MSI1 only, stop codon, and control oligos. Error bars show SEM. *G*, cumulative distribution function of UNK RiboSeq (46) fold change, log_2_, separated *via* iCLIP detection: control (*light gray*; dotted), MSI1 only (*orange*), UNK only (*green*), and shared (*purple*). Insets show significance values for all comparisons *via* two-sided KS test and corrected for multiple comparisons *via* the BH procedure. *Red* denotes significant (*p* ≤ 0.05). Values are as follows: *A* (ns), *D* (*p* ≤ 0.01), *F* (*p* ≤ 0.0001).

We used available iCLIP data for MSI1 (27) and UNK (26) and selected binding sites that contained a UAG motif in well-expressed transcripts (Experimental procedures). At the transcript level, we identified 3502 and 4074 transcripts that were bound by MSI1 or UNK, respectively (Experimental procedures). Comparing these sites across proteins, we found that 50% of these transcripts are bound by both proteins (Fig. 7*A*). Given the nucleotide-level resolution of iCLIP, we determined where on each transcript MSI1 and UNK were bound (Fig. S7*A*). Consistent with previous work, we found MSI1 bound primarily in the 3′ UTR and UNK in the coding sequence (CDS) (26, 27). Thus, even when both proteins bound the same transcript, 88% of the time they bound different motifs within that transcript (Fig. 7*B*). Interestingly, these data also show that neither protein binds UAG stop codons (26, 27), although why this is the case remains unclear.

To investigate protein-specific binding preferences and whether they are sequence or cellular-context driven, we designed a natural sequence pool containing MSI1- and UNK-specific regions as well as those that displayed evidence of being bound by both proteins (*e.g.*, shared). We also included a large set of control sequences matched for UAG content and transcript region. We were intrigued by the lack of UAG stop codon binding for both proteins (26, 27). To test if this is due to sequence context features (*i.e.*, measurable biochemical features) surrounding stop codons, we included regions containing the stop codon for transcripts that displayed evidence of binding elsewhere. Finally, as a negative control, we included mutant oligos where all occurrences of UAG were mutated to CCG to disrupt binding. This resulted in a pool of 5922 120 nt naturally derived sequences which we used to perform nsRBNS with MSI1 and UNK, separately.

We performed nsRBNS in duplicate with independent protein batches and found the replicates to be highly correlated (Fig. S7, *B* and *C*). As both MSI1 and UNK preferentially bind a UAG motif, we determined the effects of mutating this motif to a CCG and found these mutations greatly diminish binding for both proteins (Fig. S7, *D* and *E*). Further, as the number of UAG motifs increase per oligo, so do the binding enrichments of both proteins (Fig. S7, *F* and *G*). These data confirm previously reported binding studies for MSI1 and UNK (9, 21, 29) and demonstrate that this approach captures direct binding driven by known motifs embedded in the context of longer natural sequences.

Next, we defined five classes of oligos based on binding patterns *in cultured cells*: control, where no evidence of binding was observable for either protein; stop codon, stop codon regions from transcripts that MSI1 or UNK bound elsewhere on the transcript; MSI1 only or UNK only, where binding was observed for one protein but not the other; and shared, where binding was observed for both proteins at the same region on the transcript. For both proteins, we observe that both control and stop codon oligos are lowly enriched (Fig. 7, *C* and *D*). Because both sequence sets were designed to contain UAGs, this demonstrates that UAG alone is not sufficient to confer binding for either MSI1 or UNK. These *in vitro* data also suggest that the inability of MSI1 or UNK to bind the stop codon could be driven through RNA sequence, as this lack of binding is occurring outside of the cellular context. Biologically, this implies that UNK and MSI1's disfavored binding to UAG stop codon is not due to protein blocking but rather driven at the RNA sequence level.

Surprisingly, both MSI1 and UNK demonstrated equivalent enrichments for MSI1-only or UNK-only oligos (Fig. 7, *C* and *D*). That is, no *in vitro* discrimination was observed for these proteins when binding patterns are considered in aggregate, yet *in cellulo* these proteins have limited overlap in binding sites. To better understand the sequence features within these longer natural RNAs that drive binding, we used our nsRBNS data to create linear models for MSI1 and UNK to predict binding (Fig. S7, *I* and *J*). These models revealed that UAG emerged as the strongest feature for both MSI1 and UNK. Further, secondary motifs had significant contributions to enrichment prediction. Consistent with the lack of protein-specific selectivity for cellular targets *in vitro*, we found predictive features between the UNK and MSI1 models to be positively correlated (Fig. S7*K*). In contrast to the observed de-enrichment in stop codons and controls, these data imply even more complexities than sequence driving cellular RNA specificity.

Despite most cellular binding measurements (iCLIP) yielding unique sites to MSI1 or UNK, some were shared across CDS and the 3′ UTR. Shared binding sites displayed the highest enrichments for both MSI1 and UNK (Fig. 7, *C* and *D*). Thus, not only do MSI1 and UNK bind each other's unique cellular targets *in vitro* but shared cellular targets appear to have the highest affinity for both proteins. In previous work, we found that highly conserved sequences, which traditionally have some biological significance, were among the highest affinity RBP-binding sites (21). To test that shared sequence targets may be highly conserved, we measured conservation (phyloP scores (61)) of the regions included in our pool and observed that shared oligos were the highest conserved (Fig. 7*E*). Therefore, not only are both MSI1 and UNK binding these regions, they have also been retained in these genes overtime and therefore suggests the potential of regulatory importance for these RBPs, as well as others.

We hypothesized that better binding to shared targets may be due at least in part to increased accessibility of motifs. To test this, we performed *in silico* folding (53). Indeed, when comparing our oligo groups, we found that shared oligos had the lowest BPP of the cohort at and surrounding the UAG motif (Fig. 7*F*). This lower BPP (aka higher accessibility) which we identified in our posRBNS experiments very likely contributes to the binding of MSI1 and UNK to these sequences, due to less RNA structure to compete with. Further, we observed that the selected stop codon sequences had the highest BPP upstream and downstream, reinforcing our hypothesis that a lack of stop codon binding may be in part driven by RNA sequence both *in vitro* and *in cellulo* (26, 27). These data emphasize that overall motif accessibility plays an important role in RNA binding, mirroring previous studies that many RBPs preferentially bind motifs present in hairpin loops rather than base-paired stems (9, 11, 59, 62).

To understand how these observed binding patterns reflect cellular regulatory patterns, we utilized available RiboSeq data for MSI1 overexpressed in mouse cortical neuronal stem cells (47) or UNK overexpressed in HeLa cells (46) as both are translational regulators. We determined the translational impact of genes with shared or protein-specific UNK and MSI1 peaks. For UNK, we see significant repression for shared and UNK-only transcripts, much greater than for MSI1-only or control transcripts (Fig. 7*G*). Based on this initial analysis, this suggests that despite the lack of protein-specific binding *in vitro*, UNK regulates the genes to which it preferentially binds over those identified as MSI1-only peaks. For MSI1, we were unable to identify patterns that relate to binding, but this may be because MSI1's role in translation can be bidirectional (63), complicating this analysis (Fig. S7*H*). Despite the difference in cell conditions and contexts, we see that a lack of specificity from *in vitro* binding is not seen through cell-based analyses and that UNK targets are specifically repressed over the MSI1-only and control genes. This further highlights the valuable interplay between RNA sequence, which we can recapitulate outside the cell quite well, and diverse cellular factors that can drive protein-specific interactions and competition.

## Discussion

RNA–protein interactions are critical for gene regulation. Disruption of these interactions can impact disease, as greater than 50% of genetic diseases can be linked to mutations within RNA regulatory elements that often interact with RNA-binding factors (64, 65, 66). Here, we examined the RNA-binding preferences and overlapping specificities of three UAG-binding RBPs: MSI1, MSI2, and UNK. We find that these three proteins have significant primary motif overlap (Fig. 1). Overlapping motif specificity is a feature of RBPs as a class and presents an area of discrepancy between *in vitro*–binding preferences and cellular targets. That is, *in vitro*–derived motifs are unable to fully predict binding in cells (21, 67), yet their presence in RNA targets is required. Among cellular RNA targets assayed *in vitro*, binding strength correlates well with cellular regulation (9, 13, 21). Previous work has shown that features beyond short motifs including RNA structure and sequence context can provide more unique specificity profiles across RBPs, but how often these features are at play to drive binding in cells is not known.

A goal of this study was to assess RBP specificity as deeply as possible and examine binding preferences and affinities domain-by-domain to understand how each domain alone and in tandem define RNA interactions. When examining individual domains from MSI1, MSI2, and UNK, we found subtle differences that arise in sequence preference (Fig. 2) and spacing (Fig. 3). Within the RBDs, we most notably found that UNK is distinct from MSI1 and MSI2 in that while its preferred motif is UAG, ZnF1-3 actually prefers an A/U-rich motif (Fig. 2), yet as full-length RBPs, they yield highly similar binding profiles.

How highly similar proteins (*i.e.*, paralogs) often have independent RNA regulation and functions remains an intriguing question. For instance, the paralogous proteins hnRNPL and hnRNPLL both preferentially bind CA-rich motifs; however, hnRNPLL stringently prefers a spacing of two nucleotides whereas hnRNPL is more lenient (68). Importantly, we designed a high-throughput *in vitro* assay to test such differences with unprecedented depth. We included the highly conserved paralogs MSI1 and MSI2 to test if and how the RNA specificities of these proteins differ. In our own work, we observe subtle but important binding differences between MSI1 and MSI2 where both proteins prefer two-nucleotide spacing between their UAG motifs; however, MSI1 is more lenient in spacing changes and can accommodate one to five nucleotides better than MSI2 (Fig. 3). It is still unknown how these binding patterns are selected for at the amino acid level, but minute changes in disordered regions (69, 70, 71, 72, 73) and dimerization potential (74, 75) may play an important role.

The affinity of individual tandem domains is also likely to impact which targets are bound best, especially if RBDs have differences in specificity. We found that for MSI1, MSI2, and UNK, domains acting together bind RNA far better than any single domain alone, behaving as a “tandem anchoring” system (Figs. 4 and 5). We hypothesize such a model is largely applicable to other tandem-domain RBPs. Indeed, this has been previously observed for KSRP where KH3 serves as the large anchor with low μM affinity and KH1, 2, and 4 serve as stabilizing anchors enhancing the overall affinity to low nM (17). For MSI1 and UNK, we further found that the presence of more than one binding site on a given RNA can enhance binding four- to ten-fold depending on which RBP is being assayed (Figs. 4 and 5). We hypothesize that tandem RBD anchoring involving one strong and one weak RBD may be a mechanism that enables wider target selection. Furthermore, considering our domain swap results, it is highly possible that there may be a degree of stabilization within the order of the RRMs, as demonstrated by Levengood *et al.* (76). Using hnRNP A1, a similar two RRM RBP, they demonstrate that RRM1 stabilizes RRM2 through salt bridge contacts, which ultimately serve to stabilize and improve RNA binding. Through our switching of order both in the MSI1 and UNK proteins, it is probable that intraprotein contacts may have been disrupted leading to lessened binding.

A significant limitation of completely understanding RBP–RNA interactions is the often-simplistic environments present *in vitro* compared to the vast complexity in cells. Here, we attempted to increase the complexity of binding reactions by introducing a competitor protein to attempt to begin to mirror the cellular environment. For UNK with MSI1 in competition, we found many sequences are readily competed away from UNK, but UNK-preferred sequences are less competed by MSI1 (Fig. 6). Individual binding affinities must be considered when analyzing such an assay: in our own work, we found MSI1 binds much more tightly than UNK to the RNAs we tested. Therefore, we found that UNK is unable to compete RNA targets from MSI1. Even in this simplified *in vitro* system (two proteins competing for binding to a highly diverse set of sequences encompassing a full spectrum of affinities), analysis and interpretation remains a challenge. We noted that enrichments for MSI1 and UNK increased when low levels of competitor proteins are present. We interpret this to be an improvement of signal-to-noise as competitor at low levels may bind suboptimal targets, thus limiting background binding of the other factor. One might envision a similar effect in cells where target selection for a given RBP may be narrowed due to competitor proteins engaging motif-containing targets.

In an attempt to better understand cellular RNA target selection, we analyzed iCLIP data for MSI1 and UNK (26, 27). We observe that MSI1 and UNK binding defined by iCLIP reveals approximately 50% transcript-level binding conservation, but far less binding-site conservation (26, 27). Even more, the specific transcript regions that are bound are different, with UNK preferring CDS and MSI1 preferring 3′ UTRs. Despite these clear differences observed *in cultured cells*, no significant differences are observed *in vitro* with nsRBNS as UNK and MSI1 bound each other's specific sites equally. This implies that other cellular factors outside of RNA sequence and structure, which can be recapitulated through their lack of binding to stop-codon sequences, can significantly contribute to protein-specific RNA binding (Fig. 7). It is still unknown why these proteins display these transcript region specificities and is still under investigation.

Importantly, one cannot overlook cellular aspects of protein biology, including expression, localization, and complex formation, when evaluating RBP competition. On the subcellular level, as mentioned prior, these proteins can all be found in the cytoplasm (26, 77, 78), implying that at least on the cellular level, competition would be reasonable. Examination of tissue expression from the Genotype-Tissue Expression consortium (79) revealed that all three proteins selected herein have overlapping tissue expression patterns, but this is not ubiquitously true: MSI1 is primarily expressed in the testes, MSI2 in ovaries, and UNK in the cervix. Further, many tissues demonstrate similar expression levels between two of the three of these proteins. For example, MSI1 and UNK have similar expression levels across many brain tissues, while MSI2 is elevated even higher. How these similarities in expression across tissues ultimately contribute to the cellular binding environment and associated phenotypes remains an important research challenge and is a future direction driven through our approach.

Previous work by Lang *et al.* demonstrated with enhanced CLIP data (10) that RBPs often form complexes to bind and regulate their RNA targets (67). UNK is known to associate to ribosomes (46) and this association may contribute to higher CDS binding in cultured cells than would be predicted based on *in vitro* data (21). However, how much this association affects UNK's binding patterns and if there's any binding influence between MSI1 and UNK remains to be seen. Other proteins have been found to interact with ribosomes, transcription machinery, and degradation machinery (80, 81, 82, 83, 84, 85, 86, 87, 88, 89). For instance, the LSm complex, which is essential for RNA degradation (90), is recruited to histone mRNA by SLBP (91). Further, several splicing factors are recruited to pre-mRNA by RNA polymerase II (92, 93, 94), which may be critical for their interactions with nascent RNA and splice sites.

The work described here presents a detailed, unbiased approach to fully catalog the RNA-binding preferences of multidomain RBPs to better understand how individual RBDs specify their target RNAs in cultured cells. Understanding complex formation in regard to RNA targeting remains an important challenge. Future work will involve building models starting from *in vitro* unbiased data where individual domains are cataloged independently and specificity is well understood to develop a model for RBP-RNA recognition. However, adding cellular features such as protein–protein interactions, RBP localization, and transcript region preferences will be essential to grow our understanding of RBP binding and regulation in cells. We believe this novel, high-throughput approach provides more detail and analytical depth than previously possible, allowing for a more complete understanding of RNA-protein interactions.

## Experimental procedures

### Cloning of pGEX constructs

pGEX-GST-SBP-MSI1 (mouse) (9) and pGEX-GST-SBP-UNK (30–357) (9) were gifted from Chris Burge (MIT). pGEX-GST-SBPUNK ZnF1-3 and pGEX-GST-SBP-UNK ZnF4-6 have been previously published by our group (21). DNA for MSI2 was ordered from Twist Biosciences. RBD sequences were selected for MSI1 as published by Iwaoka *et al.* (30) and MSI2 as published by Lan *et al.* (74). Domain annotations (Table S1) were verified with UniProt (33) and InterPro (51). Primers for insert amplification and plasmid construction were synthesized by Integrated DNA Technologies (IDT; Table S2).

Standard PCR was used to amplify plasmid inserts; restriction enzymes (New England Biolabs, NEB) were used to cleave plasmid backbones, and In-Fusion (Takara Bio) cloning was utilized according to manufacturer recommendations. Following in-fusion cloning, plasmids were transformed into Stellar competent cells (Takara Bio), then miniprepped (Qiagen) and sequence verified *via* Sanger sequencing (Genewiz). Relevant plasmid information for this study (backbone, restriction sites used, insert, *etc.*) can be found in Table S3.

### Expression and purification of recombinant proteins

SBP-MSI1 (mouse) (9), SBP-MSI2, SBP-UNK (30–357) (9), Flag-MSI1 (mouse), HIS-GST-UNK (30–357), SBP-MSI1 RRM1, SBP-MSI1 RRM2, SBP-MSI2 RRM1, SBP-MSI2 RRM2 SBP-UNK ZnF1-3 (21), and SBP-UNK ZnF4-6 (21) were purified as previously described (21) with slight modifications. Rosetta *E. coli* competent cells (Novagen) were used for all purifications. Transformed cultures were grown in LB media at 37 °C until an A of 0.8 was reached, then induced with 0.5 mM IPTG (Thermo Fisher Scientific) at 16 °C for 24 h. Cultures were centrifuged at 4 °C for 15 min at 4000*g* to harvest cells, resuspended in lysis buffer (200 mM NaCl, 5 mM DTT, 50 mM Hepes, 3 mM MgCl_2_, 2 mM PMSF, 1 PierceTM protease inhibitor mini tablet/2 L; Thermo Fisher Scientific), sonicated, then incubated at 25 °C for 30 min with 500 units/1 L culture Benzonase Nuclease (Sigma-Aldrich) and 5 units/1 L RQ1 RNase-free DNase (Promega). Lysate was clarified *via* centrifugation at 17,800*g* for 30 min at 4 °C.

Pierce Glutathione Agarose (Thermo Fisher Scientific) was used for protein purification. Beads were equilibrated in low salt buffer (300 mM NaCl, 50 mM Hepes) prior to 1 h incubation with lysate at 4 °C. Recombinant protein-bound beads were washed in low salt buffer and high salt buffer (1 M NaCl, 50 mM Hepes) prior to incubation overnight at 4 °C with 1:50 PreScission Protease (Cytiva) in cleavage buffer (20 mM Hepes, 100 mM NaCl, 5 mM DTT, 10% glycerol, 0.01% Triton X-100). For SBP-MSI2, buffers were as follows due to predicted disordered regions: lysis buffer (200 mM NaCl, 5 mM DTT, 50 mM Hepes, 3 mM MgCl_2_, 2 mM PMSF, 1 Pierce protease inhibitor mini tablet/2 L, 1% Triton X-100), low salt buffer (300 mM NaCl, 50 mM Hepes, 5 mM EDTA, 0.1% Triton X-100), and high salt buffer (1 M NaCl, 50 mM Hepes, 5 mM EDTA, 0.1% Triton X-100).

Proteins were eluted off the beads in excess cleavage buffer, concentrated *via* spin column (Vivaspin; Cytiva), and quality controlled *via* Pierce 660 nm assay (Thermo Fisher Scientific) and SDS-PAGE (4–12% gradient). For SBP-MSI1 (mouse), SBP-MSI2, SBP-UNK (30–357), Flag-MSI1 (mouse), HIS-GST-UNK (30–357), size-exclusion chromatography (Superdex 200 Increase 10/300 Gl; Cytiva) was used for further purification. Fractions were separated and collected in size-exclusion buffer (20 mM Hepes, 1 M NaCl, 10 mM DTT, 0.01% Triton X-100), and relevant fractions were pooled.

### Design and *in vitro* transcription of RBNS pools

20mer RBNS and posRBNS DNA pool was synthesized by IDT (Table S4) and was *in vitro* transcribed using T7 RiboMAX Express large scale RNA kit (Promega) according to manufacturer protocols.

### Random RBNS

RBNS was performed as previously described (9, 49) with slight modifications. Recombinant SBP-tagged protein was incubated at 4 °C for 30 min with MyOne Streptavidin T1 Dynabeads (Thermo Fisher Scientific) at multiple concentrations (SBP-MSI2: 50, 250 nM; SBP-MSI1 RRMs: 250, 500, 1000 nM; SBP-MSI2 RRMs: 250, 500, 1000 nM; SBP-UNK ZnF4-6: 250, 500, 1000 nM) in RBNS-binding buffer (25 mM Tris pH 7.5, 150 mM KCl, 3 mM MgCl_2_, 0.01% triton-X 100, 500 μg/ml BSA, 20 units/ml SUPERase·In (Thermo Fisher Scientific)). Unbound protein was removed, protein-bead complexes were resuspended in RBNS-binding buffer, then incubated with 1 μM 20mer RBNS RNA at 4 °C for 1 h. Protein–RNA-bead complexes were washed in RBNS wash buffer (25 mM Tris pH 7.5, 150 mM KCl, 0.01% Triton X-100, 20 units/ml SUPERase·In) prior to elution at 60 °C in RBNS elution buffer (0.1% SDS, 0.3 mg/ml proteinase K (Thermo Fisher Scientific)) for 30 min. Elution was repeated and elutions were pooled prior to phenol chloroform extraction.

Eluted RNA was reverse transcribed with Superscript IV (Invitrogen) with RBNS RT primer (Supporting Information) and PCR amplified *via* Phusion DNA polymerase (New England Biolabs) with RBNS index primers and RBNS reverse primer (Supporting Information). Sequencing was performed on an Illumina NextSeq 1000. 6mer enrichments were calculated as the normalized count (frequency) of each 6mer within the bound pool *versus* the frequency in the input pool as reported previously (9). Full binding reactions were performed in duplicate with independent protein batches, and the replicate enrichments were averaged. Logos were generated with the top 15 6mers, aligning to the top enriched 6mer. Fastqs for SBP-MSI1 and SBP-UNK were obtained from ENCODE (9) (MSI1: ENCSR329RIP, UNK: ENCSR497VCL) and processed as detailed above. Fastqs for SBP-UNK ZnF1-3 and one replicate for SBP-UNK ZnF4-6 were obtained from GEO (21) (GEO; GSE262560) and processed as detailed above.

Final logos were plotted in RStudio (95) (version 4.3.1) with positional weight matrices fed into ggseqlogo (96). Additional R packages “data.table (97)” (version 1.16.0), “ggnewscale (98)” (version 0.5.0), “dplyr (99)” (version 1.1.4), “ggplot2 (100)” (version 3.5.1), “ggpubr (101)” (version 0.6.0), “ggrepel (102)” (version 0.9.6), “stringr (103)” (version 1.5.1) were used for data analysis and plotting.

### Positional RBNS

posRBNS was performed similarly to RBNS (see above) with slight modifications. SBP-MSI1, SBP-MSI2, and SBP-UNK were incubated at 1, 10, 100, and 1000 nM with 1 μM posRBNS RNA. Kmer enrichments were calculated positionally upstream and downstream of the central UAG where reads including an additional UAG outside of the central or the counted position were excluded. Logos were generated separately upstream and downstream with the top five 4mers and manually aligned. Final logos were plotted in RStudio (95) with R package seqLogo (104) (version 1.66.0). Additional R packages “dplyr (99)” (version 1.1.4), “ggplot2 (100)” (version 3.5.1), “ggpubr (101)” (version 0.6.0), “ggrepel (102)” (version 0.9.6), and “stringr (103)” (version 1.5.1) were used as needed.

### PosRBNS and iCLIP pattern analysis

MSI1 iCLIP-seq data from glioblastoma was downloaded from Uren *et al.* (27) (GSE68800), MSI2 iCLIP-seq in K562 cells overexpressing MSI2 was downloaded from Karmakar *et al.* (28) (GSE93210), and UNK iCLIP-seq data in HeLa cells overexpressing UNK or SH-SY5Y cells was downloaded from Murn *et al.* (26) (E-MTAB-2279). As needed, peaks were converted from hg18 (MSI1) or hg19 (MSI2 and UNK) to hg38 using liftOver (105) (version 1.24.0) in RStudio(RStudio 2020) with liftOver chains obtained from UCSC. As all three proteins are cytoplasmically localized (26, 77, 78), only peaks overlapping with exons were including. This was done with AnnotationHub (106) (version 3.8.0). R package “ensembldb (107)” (version 2.24.1) was also used. Further, only one-to-one human-to-mouse orthologous genes with >5 TPM expression in glioblastoma, MSI2-overexpressing K562, SH-SY5Y, or UNK-overexpressing HeLa cells from the associated studies were included.

Peaks were expanded to 100 base pairs and sequences were obtained with function “getSeq” using BSgenome.Hsapiens.UCSC.hg38(108) (version 1.4.5) and filtered for “TAG” as both proteins primarily require a TAG to bind (9, 26, 27, 29). Peaks were centered around a “TAG” motif and collapsed to 33 nucleotides. For MSI1 and MSI2, replicates were concatenated, and for UNK, both cell types were concatenated prior to downstream processing. Single protein peaks were sorted and merged with BEDTools (109) (version 2.30). Peaks were expanded back out to 100 nucleotides to recenter at “TAG” following the merge, then trimmed back down to 33 nucleotides with a central “TAG” motif.

We annotated the genomic region of each peak according to the GENCODE (v34). Then we generated control unbound regions based on these genomic regions. For UTR regions, the control was randomly sampled from the same region of same gene. For CDS regions, the control was chosen among the regions that preserved the distance from the nearest splice sites.

Bipartite patterns were identified and counted in posRBNS and iCLIP reads and normalized for library size then to controls (input RNA for posRBNS and control regions for iCLIP).

### Fluorescence polarization

3′ 6-FAM fluorescently-tagged RNA was ordered from IDT (Supporting Information). Serial diluted recombinant SBP-tagged protein was incubated with 5 nM RNA in FP binding buffer (20 mM Hepes, 5 mM DTT, 137.5 mM NaCl, 0.01% Triton X-100, 10 ng/μl BSA, 2 units/ml SUPERase⋅In) at 4 °C for 15 min. For positional FP, 50 nM untagged random RNA (N_16_) was added to increase RNA competition. FP was measured at 25 °C with a CLARIOstar plate reader (BMG Labtech). FP binding assays were performed in triplicate with independent protein batches. Where relevant, delta FP was calculated to account for minimum and maximum FP. All data plots are available in Supporting Data 1. Data were fit in GraphPad Prism to a single site–binding model to determine a K_d_.

### Natural sequence RBNS

nsRBNS was performed similarly to RBNS (see above) with slight modifications. SBP-MSI1 and SBP-UNK were incubated at 100 nM with 1 μM nsRBNS RNA. SBP-UNK ZnF1-3 and SBP-UNK ZnF4-6 were assayed at 500 nM. The UNK-centric nsRBNS pool was similar to previously published (21), but with different control sequences.

### nsRBNS mapping and enrichment analysis

Reads were trimmed using fastx_toolkit (110) (version 0.0.14) as needed prior to mapping with STAR (111) (version 2.7.10 b) with parameters set to –outFilterMultimapNMax 1 and –outFilterMismatchNmax 1. Seqk (112) (version 2.3.0) was used to trim input fasta file. Enrichment was calculated as the frequency of the full-length sequence in the bound pool over the frequency in the input pool. For MSI1 and UNK nsRBNS, reads with less than 25 counts were excluded. Final sequences, enrichments, and relevant oligo information are included in Supp. Data 2 to 5. RStudio (95) was used for data analysis and plotting as needed with packages “ggplot2 (100)” (version 3.5.1), “ggpubr (101)” (version 0.6.0), and “stringr (103)” (version 1.5.1).

### Competition posRBNS

Competition posRBNS was performed similarly to posRBNS (see above) with slight modifications. SBP-tagged proteins were held at 100 nM. Following removal of unbound SBP-tagged protein, untagged protein (UNK or Flag-MSI1) was added at 10, 100, or 1000 nM to incubate with protein–bead-RNA complexes for 1 h. Elution, RT, and PCR was performed as described above. Enrichments were calculated positionally as detailed above, then normalized to 0 nM antagonist protein. R packages “dplyr (99)” (version 1.1.3), “ggplot2 (100)” (version 3.5.1), and “ggpubr (101)” (version 0.6.0) were used as needed.

### iCLIP and peak overlap analysis

MSI1 and UNK iCLIP-seq data was processed as described above with slight modifications. Peaks were expanded out to 100 base pairs prior to concatenating replicates. Following merge, MSI1 and UNK peaks were intersected with BEDTools (109) to determine overlapping regions. R package “VennDiagram (113)” (version 1.7.3) was used to create publication-quality Venn diagram. Multimapping peaks were removed. Peaks were expanded to 250 base pairs and aligned at a central “TAG.” All overlapping peaks were included. For nonoverlapping peaks, top-scoring iCLIP peaks were preferred with approximately 50:50 distribution between CDS and 3′ UTR.

All CDS and 3′ UTR sequences were downloaded from Ensembl BioMart (114). Stop codon–centered sequences were selected for all overlapping peaks as well as top-scoring MSI1- or UNK-bound genes. Controls were selected randomly from genes not demonstrating MSI1 or UNK binding *via* iCLIP and were matched for TAG content with bound regions. For iCLIP processing and pool assembly, R packages “AnnotationHub (106)” (version 3.8.0), “dplyr (99)” (version 1.1.4), “ensembldb (107)” (version 2.24.1), “seqParser (115)” (version 0.1.0), “stringr (103)” (version 1.5.1), and “tidyr (116)” (version 1.3.1) were used.

### Linear modeling

nsRBNS data for UNK and MSI were used for linear models. To overlay structure and sequence, we used RNAfold (53) to extract base pair probabilities for each sequence. We next assigned kmers within a sequence into “more structured” and “less structured” based on the mean BPP of the nucleotides comprising each kmer. If a given kmer had a BPP greater than the median of all kmers of that sequence (for all nsRBNS sequences), then we assigned it as “more structured,” if it had a lower BPP than the median it was assigned as “less structured.” This was done since kmers have varying distributions of BPP (*e.g.* GC-rich kmers have higher BPP). We used 3mers to build the linear model, then performed stepwise model selection by Akaike information criterion, which resulted in 84 features for MSI1 and 76 features for UNK. For the correlation between UNK and MSI1 models, only the coefficients of overlapping features were used. R packages “cowplot (117)” (version 1.1.3), “flextable (118)” (version 0.9.6), “grid” (version 4.3.1), and “MASS (119)” (version 7.3.60) were used as needed.

## Data availability

Enrichments generated in the study for RBNS, posRBNS, Competition posRBNS, and nsRBNS have been deposited in Gene Expression Omnibus (GEO) under accession code GSE280076. Raw fastQ files are also available in GEO. Additional data used in this study have been accessed through the following sources: MSI1 iCLIP-seq data (27): GSE68800; MSI2 iCLIP-seq data (28): GSE93210; UNK iCLIP-seq data (26): E-MTAB-2279; MSI1 RBNS (9): ENCSR329RIP; UNK RBNS (9): ENCSR497VCL; UNK ZnF RBNS (21): GSE262560.

## Code availability

All scripts for data processing and figure generation are available at https://github.com/DominguezRNAGroup/Tandem_RBD_Analysis.

## Supporting information

This article contains supporting information (9, 21, 26, 27, 29, 30, 31, 47, 49, 52).

## Conflict of interests

The authors declare that they have no conflicts of interests with the contents of this article.

## Supporting information

Supp_compiled

Supp_figures_compiled
