# Supplementary material for: Dissecting RNA selectivity mediated by tandem RNA-binding domains
Source: J Biol Chem. 2025 Mar 20;301(5):108435. doi: 10.1016/j.jbc.2025.108435 (PMC12136788; doi:10.1016/j.jbc.2025.108435)
Supplement: Supp_compiled [file mmc1.docx]

**Index of Supplementary Information for Harris *et al*.**

Supplementary Figures

- Supp. Figure 1 🡪 relates to Figure 1: Motif preferences for full-length MSI1, MSI2, and UNK as determined via RBNS
- Supp. Figure 2 🡪 relates to Figure 2: Motif preferences for individual RBDs of MSI1, MSI2, and UNK as determined via RBNS
- Supp. Figure 3 🡪 relates to Figure 3: Secondary motif, spacing, and base pair probability preferences for MSI1, MSI2, and UNK as determined via posRBNS
- Supp. Figure 4 🡪 relates to Figure 4: Affinity of MSI1 and MSI2 for polyN RNA
- Supp. Figure 5 🡪 relates to Figure 5: Combinatorial affinity of UNK for positional motifs
- Supp. Figure 6 🡪 relates to Figure 6: Competition of secondary motifs between MSI1 and UNK as determined via posCompRBNS
- Supp. Figure 7 🡪 relates to Figure 7: Analysis of MSI1 and UNK competition potential *in vivo* and *in vitro*

Supplementary Tables

- Supp. Table 1 🡪 Domain regions for selected proteins
- Supp. Table 2 🡪 Primer sequences for cloning of recombinant protein constructs
- Supp. Table 3 🡪 Plasmid design for relevant plasmids
- Supp. Table 4 🡪 Relevant oligo information for 20mer and posRBNS RNA
- Supp. Table 5 🡪 Primer sequences for RBNS reverse transcription and PCR
- Supp. Table 6 🡪 Sequences for synthetic fluorescence polarization RNA oligos

Supplementary References

Supplementary Download Items

- Supp. Data 1 🡪 relates to Figures 4 and 5 and Supp. Figures 4 and 5: Data points plotted for all fluorescence polarization experiments
- Supp. Data 2 🡪 relates to Figure 5: UNK ZnF1-3 nsRBNS data table used for all analyses with sequences, relevant iCLIP information, enrichment values, relevant sequence information, and relevant oligo information
- Supp. Data 3 🡪 relates to Figure 5: UNK ZnF4-6 nsRBNS data table used for all analyses with sequences, relevant iCLIP information, enrichment values, relevant sequence information, and relevant oligo information
- Supp. Data 4 🡪 relates to Figure 7: MSI1 overlapping nsRBNS data table used for all analyses with sequences, relevant iCLIP information, enrichment values, relevant sequence information, and relevant oligo information
- Supp. Data 5 🡪 relates to Figure 7: UNK overlapping nsRBNS data table used for all analyses with sequences, relevant iCLIP information, enrichment values, relevant sequence information, and relevant oligo information

**SUPPLEMENTAL FIGURE LEGENDS**

**Supp. Figure 1. Motif preferences for full-length MSI1, MSI2, and UNK as determined via RBNS.** A) Bar plot of log_2_ RBNS enrichments of top 6mers for MSI1, MSI2, and UNK at all assayed concentrations. MSI1 and UNK data assayed by Dominguez *et al.^1^*.

**Supp. Figure 2. Motif preferences for individual RBDs of MSI1, MSI2, and UNK as determined via RBNS.** A-C) Bar plot of log_2_ RBNS enrichments of top 6mers for A) MSI1 RRM1 and MSI1 RRM2, B) MSI2 RRM1 and MSI2 RRM2, and C) UNK ZnF1-3 and UNK ZnF4-6 at all assayed concentrations. One replicate of UNK ZnF1-3 and UNK ZnF4-6 assayed by Harris *et al.^2^*. top ten 3mers and enrichment values as determined by iterative *k*mer analysis^1,3^ included. D) Alignment of MSI1 RRM1 and MSI1 RRM2 as analyzed by BLAST^4^. RNA-contacting residues as determined by NMR structures^5,6^ conserved across RRMs highlighted in red with similar amino acids highlighted in blue. E) Alignment of UNK ZnF1-3 and UNK ZnF4-6 as analyzed by BLAST^4^. RNA-contacting residues as determined by crystal structures^7^ across domains highlighted in red with similar amino acids highlighted in blue and non-conserved residues highlighted in orange for ZnF1-3 and green for ZnF4-6.

**Supp. Figure 3. Secondary motif, spacing, and base pair probability preferences for MSI1, MSI2, and UNK as determined via posRBNS.** A) Heat map of the log_2_ posRBNS enrichment of all 3mers for MSI1. B) Heat map of the base pair probability of all 3mers for MSI1. C) Heat map of the log_2_ posRBNS enrichment of all 3mers for MSI2. D) Heat map of the base pair probability of all 3mers for MSI2. E) Heat map of the log_2_ posRBNS enrichment of all 3mers for UNK. F) Heat map of the base pair probability of all 3mers for UNK. G-I) Line plot of the base pair probability of CCC for H) MSI1, I) MSI2, and J) UNK.

**Supp Figure 4. Affinity of MSI1 and MSI2 for polyN RNA.** A) Fluorescence polarization binding curves (n=3) for full-length MSI1 (orange circle), MSI1 RRM1 (light orange square), and MSI1 RRM2 (dark orange triangle) incubated with a polyN oligo. Data are presented as mean values +/- SD. B) Fluorescence polarization binding curves (n=3) for full-length MSI2 (teal circle), MSI2 RRM1 (light teal square), and MSI2 RRM2 (dark teal triangle) incubated with a polyN oligo. Data are presented as mean values +/- SD. C) Fluorescence polarization binding curves (n=3) for all RNA-binding domains of UNK (green circle), UNK ZnF1-3 (light green square), and UNK ZnF4-6 (dark green triangle) incubated with a polyN oligo. Data are presented as mean values +/- SD.

**Supp. Figure 5. Combinatorial affinity of UNK for positional motifs.** A) Fluorescence polarization binding curves (n=3) for UNK incubated with a polyU oligo (blue circle) or a polyC oligo (yellow square). Data are presented as mean values +/- SD. B) Correlation plot log_2_ ZnF1-3 nsRBNS enrichments versus log_2_ UNK nsRBNS enrichments. Pearson’s correlation coefficient and p value included. C) Correlation plot log_2_ ZnF4-6 nsRBNS enrichments versus log_2_ UNK nsRBNS enrichments. Pearson’s correlation coefficient and p value included. D) Correlation plot log_2_ ZnF1-3 nsRBNS enrichments versus log_2_ ZnF4-6 nsRBNS enrichments. Pearson’s correlation coefficient and p value included.

**Supp. Figure 6. Competition of secondary motifs between MSI1 and UNK as determined via posCompRBNS.** A-B) Log_2_ posComp enrichments of A) CCC or B) AUA with UNK and MSI1 in competition at 0, (green), 10 (light orange), 100 (orange), or 1000 nM (dark orange). Only oligos with a single UAG were considered. C-E) Log_2_ posComp enrichments of C) CCC, D) UAG, E) AUA with MSI1 and UNK in competition at 0, (orange), 10 (light green), 100 (green), or 1000 nM (dark green). F) Rank depletion of the top ten UAGN_7_ 10mers for MSI1 with UNK in competition. Arrows denote changes to rank with grey denoting maintenance within top ten and red denoting loss of top ten rank.

**Supp. Figure 7. Analysis of MSI1 and UNK competition potential *in vivo* and *in vitro*.** A) Diagram of iCLIP peak overlap analysis method between MSI1 (from glioblastoma)^8^ and UNK (from SH-SY5Y or UNK-overexpressing HeLa)^9^. B-C) Correlation plot of two experimental B) MSI1 or C) UNK overlap pool nsRBNS replicates. Pearson’s correlation coefficient and p value included. D-E) Scatter plot of log_2_ D) MSI1 or E) UNK overlap pool nsRBNS enrichments of wild-type (Y-axis) versus total motif mutant (X-axis) oligos. Log_2_ change in enrichment (wt-mut) was calculated for each sequence pair: > 0.5 defined as bound better in wt (blue), < -0.5 defined as bound better in mut (red), 0 +/- 0.5 defined as similar binding (grey). Significance determined via paired, one-sided Wilcoxon test. F-G) Cumulative distribution function of log_2_ F) MSI1 or G) UNK overlap pool nsRBNS enrichment of all oligos separated by UAG motif content. Insets show significance values for all comparisons via two-sided KS test and corrected for multiple comparisons via the BH procedure. Red denotes significant (p≤0.05), and grey denotes nearing significant (p≤0.1). Values are as follows: a (ns), b (p≤0.1), d (p≤0.01), e (p≤0.001), f (p≤0.0001). H) Cumulative distribution function of MSI1 RiboSeq^10^ absolute fold change, log_2_, separated via iCLIP detection: control (light grey; dotted), MSI1 only (orange), UNK only (green), and shared (purple). Insets show significance values for all comparisons via two-sided KS test and corrected for multiple comparisons via the BH procedure. Red denotes significant (p≤0.05). Values are as follows: a (ns), c (p≤0.05), d (p≤0.01), e (p≤0.001), f (p≤0.0001). I-J) Linear modeling of nsRBNS sequences for I) MSI1 and J) UNK. (left) Plot of linear model coefficients for top motifs as defined by RBNS, colored by -log_10_ p. (center) Table of linear model coefficients and -log_10_ p for top motifs. (right) Correlation of fitted log_2_ nsRBNS enrichment via linear model versus observed log_2_ nsRBNS enrichment. K) (left) Correlation of fitted log_2_ nsRBNS enrichment for UNK based on MSI1 model versus observed log_2_ nsRBNS enrichment. (center) Correlation of fitted log_2_ nsRBNS enrichment for MSI1 based on UNK model versus observed log_2_ nsRBNS enrichment. (right) Correlation of linear model coefficients for UNK and MSI1 models.

**SUPPLEMENTARY TABLES**

**Supp. Table 1. Domain regions for selected proteins**

| Protein | Domain | Start | Stop |
| --- | --- | --- | --- |
| MSI1 | RRM | 20 | 103 |
| MSI1 | RRM | 109 | 191 |
| MSI2 | Disordered | 1 | 28 |
| MSI2 | RRM | 21 | 111 |
| MSI2 | RRM | 110 | 187 |
| MSI2 | Disordered | 264 | 286 |
| UNK | Disordered | 1 | 24 |
| UNK | Disordered | 1 | 24 |
| UNK | ZnF | 34 | 73 |
| UNK | ZnF | 84 | 113 |
| UNK | ZnF | 124 | 154 |
| UNK | ZnF | 215 | 240 |
| UNK | Disordered | 239 | 265 |
| UNK | ZnF | 251 | 285 |
| UNK | ZnF | 293 | 321 |
| UNK | Disordered | 324 | 343 |
| UNK | Coil | 647 | 681 |
| UNK | Coil | 696 | 723 |

**Supp. Table 2. Primer sequences for cloning of recombinant protein constructs**

| Primer name | Sequence |
| --- | --- |
| pGEX-GST-Flag-MSI1 (mouse) forward primer / pGEX-GST-SBP-MSI1 swap (mouse) forward primer | GCGTGAACCGGGATCCATGGAGACTGACGCGC |
| pGEX-GST-Flag-MSI1 (mouse) reverse primer | ACGATGCGGCCGCCTATCAGTGGTACCCATTG |
| pGEX-GST-SBP-MSI1 swap (mouse) mid1 forward primer | CTCGCCGCACGACCCCAAGAAGATCTTCGTGG |
| pGEX-GST-SBP-MSI1 swap (mouse) mid1 reverse primer | CCACGAAGATCTTCTTGGGGTCGTGCGGCGAG |
| pGEX-GST-SBP-MSI1 swap (mouse) mid2 forward primer | AAAGGAGGTGATGTCCATGGTCACTCGGACGTGCAAGATGTTCATCG |
| pGEX-GST-SBP-MSI1 swap (mouse) mid2 reverse primer | CGATGAACATCTTGCACGTCCGAGTGACCATGGACATCACCTCCTTT |
| pGEX-GST-SBP-MSI1 swap (mouse) mid3 forward primer | AAGAGCACAGCCTAAGCCGACAGGCTCAGCCC |
| pGEX-GST-SBP-MSI1 swap (mouse) mid3 reverse primer | GGGCTGAGCCTGTCGGCTTAGGCTGTGCTCTT |
| pGEX-GST-SBP-MSI1 swap (mouse) reverse primer | GTCACGATGCGGCCGCTCAGTGGTACCCATTG |
| pGEX-GST-SBP-MSI1 RRM1 (mouse) forward primer | GCGTGAACCGGGATCCTGCAAGATGTTCATCG |
| pGEX-GST-SBP-MSI1 RRM1 (mouse) reverse primer | ACGATGCGGCCGCTCACTTAGGCTGTGCTCTT |
| pGEX-GST-SBP-MSI1 RRM2 (mouse) forward primer | GCGTGAACCGGGATCCAAGAAGATCTTCGTGG |
| pGEX-GST-SBP-MSI1 RRM2 (mouse) reverse primer | ACGATGCGGCCGCTCAGGACATCACCTCCTTT |
| pGEX-GST-SBP-MSI2 forward primer | GCGTGAACCGGGATCCATGGAGGCAAATGGGA |
| pGEX-GST-SBP-MSI2 reverse primer | GTCACGATGCGGCCGCTCAATGGTATCCATTT |
| pGEX-GST-SBP-MSI2 RRM1 forward primer | GCGTGAACCGGGATCCGGTAAAATGTTTATCG |
| pGEX-GST-SBP-MSI2 RRM1 reverse primer | ACGATGCGGCCGCTCATTTCTTTGTTCTTGTG |
| pGEX-GST-SBP-MSI2 RRM2 forward primer | GCGTGAACCGGGATCCAAGAAAATATTTGTAG |
| pGEX-GST-SBP-MSI2 RRM2 reverse primer | ACGATGCGGCCGCTCACGGCTGAGCTTTCTTA |
| pGEX-GST-SBP-UNK swap forward primer / pGEX-GST-SBP-UNK dual forward primer | GCGTGAACCGGGATCCCCTCGGTGGCAAGAGA |
| pGEX-GST-SBP-UNK swap mid1 forward primer / pGEX-GST-SBP-UNK dual mid1 forward primer | TTCCTCAGCTGTGTCCGGCCAGACCACGGTAG |
| pGEX-GST-SBP-UNK swap mid1 reverse primer / pGEX-GST-SBP-UNK dual mid1 reverse primer | CTACCGTGGTCTGGCCGGACACAGCTGAGGAA |
| pGEX-GST-SBP-UNK swap mid2 forward primer | GATCCTCAGCGAGGAGCCGCAGCACTACACGT |
| pGEX-GST-SBP-UNK swap mid2 reverse primer | ACGTGTAGTGCTGCGGCTCCTCGCTGAGGATC |
| pGEX-GST-SBP-UNK swap mid3 forward primer | GGAGGCCTTGCAGAATAGCCCCACCCAGCCAG |
| pGEX-GST-SBP-UNK swap mid3 reverse primer | CTGGCTGGGTGGGGCTATTCTGCAAGGCCTCC |
| pGEX-GST-SBP-UNK swap reverse primer / pGEX-GST-SBP-UNK dual reverse primer | ACGATGCGGCCGCTCAGCTCACAGGCACCGAG |

**Supp. Table 3. Plasmid design for relevant plasmids**

| Backbone | Insert | Tag(s) | Restriction Sites |
| --- | --- | --- | --- |
| pGEX-6P-1 | MSI1 (mouse) | GST, Flag | XhoI, NotI |
| pGEX-6P-1 | MSI1 swap (mouse; 1-19,109-191,104-108,20-103,192-362) | GST, SBP | BamHI, NotI |
| pGEX-6P-1 | MSI1 RRM1 (mouse; 20-103) | GST, SBP | BamHI, NotI |
| pGEX-6P-1 | MSI1 RRM2 (mouse; 109-191) | GST, SBP | BamHI, NotI |
| pGEX-6P-1 | MSI2 | GST, SBP | BamHI, NotI |
| pGEX-6P-1 | MSI2 RRM1 (21-111) | GST, SBP | BamHI, NotI |
| pGEX-6P-1 | MSI2 RRM2 (110-187) | GST, SBP | BamHI, NotI |
| pGEX-6P-1 | UNK (30-357) | HIS, GST | XhoI, NotI |
| pGEX-6P-1 | UNK swap (204-335,175-203,31-174,336-357) | GST, SBP | BamHI, NotI |
| pGEX-6P-1 | UNK dual (204-335, 165-357) | GST, SBP | BamHI, NotI |

**Supp. Table 4. Relevant oligo information for 20mer and posRBNS RNA**

| Oligo Name | Sequence |
| --- | --- |
| 20merRBNS DNA template | CCTTGACACCCGAGAATTCCANNNNNNNNNNNNNNNNNNNNGATCGTCGGACTGTAGAACTCCCTATAGTGAGTCGTATTA |
| posRBNS DNA template | CCTTGACACCCGAGAATTCCANNNNNNNNNNNNNNNCTANNNNNNNNNNNNNNNGATCGTCGGACTGTAGAACTCCCTATAGTGAGTCGTATTA |
| 20merRBNS RNA | GAGUUCUACAGUCCGACGAUCNNNNNNNNNNNNNNNNNNNNUGGAAUUCUCGGGUGUCAAGG |
| posRBNS RNA | GAGUUCUACAGUCCGACGAUCNNNNNNNNNNNNNNNUAGNNNNNNNNNNNNNNNUGGAAUUCUCGGGUGUCAAGG |

**Supp. Table 5. Primer sequences for RBNS reverse transcription and PCR**

| Primer name | Sequence |
| --- | --- |
| RBNS RT primer | TCCTTGGCACCCGAGAATTCCANNNNNNNNTGATGCTCAATCCTCTGTTG |
| RBNS index primer | CAAGCAGAAGACGGCATACGAGAT[N_6_ barcode]GTGACTGGAGTTCCTTGGCACCCGAGAATTCCA |
| RBNS reverse primer | AATGATACGGCGACCACCGAGATCTACACGTTCAGAGTTCTACAGTCCGA |

**Supp. Table 6. Sequences for synthetic fluorescence polarization RNA oligos**

| Oligo name | Sequence |
| --- | --- |
| Mono-UAG FP RNA | UAGUUUAGUUUAGUU /6-FAM/ |
| Dual-UAG FP RNA | UUAGUUAGUU /6-FAM/ |
| Tri-UAG FP RNA | AAAAAAAAUUAGAAAAAA /6-FAM/ |
| CCC-UAG FP RNA | CCCCCCAAUUAGAAAAAA /6-FAM/ |
| UAG-AUA FP RNA | AAAAAUUAGUUAAUA /6-FAM/ |
| CCC-UAG-AUA FP RNA | CCCCCCAAUUAGUUAAUA /6-FAM/ |
| polyN FP RNA | NNNNNNNNNNNNNNNN /6-FAM/ |
| polyC FP RNA | CCCCCCCCCCCCCCCC /6-FAM/ |
| polyU FP RNA | UUUUUUUUUUUUUUUU /6-FAM/ |

**SUPPLEMENTARY REFERENCES**

1. Dominguez, D. *et al.* Sequence, structure, and context preferences of human RNA binding proteins. *Mol Cell* **70**, 854–867 (2018).

2. Harris, S. E. *et al.* Understanding species-specific and conserved RNA-protein interactions in vivo and in vitro. *Nat Commun* **15**, 8400 (2024).

3. Lambert, N. *et al.* RNA Bind-n-Seq: Quantitative Assessment of the Sequence and Structural Binding Specificity of RNA Binding Proteins. *Mol Cell* **54**, 887–900 (2014).

4. Altschul, S. F., Gish, W., Miller, W., Myers, E. W. & Lipman, D. J. Basic local alignment search tool. *J Mol Biol* **215**, 403–410 (1990).

5. Iwaoka, R. *et al.* Structural Insight into the Recognition of r (UAG) by Musashi-1 RBD2, and Construction of a Model of Musashi-1 RBD1-2 Bound to the Minimum Target RNA. *Molecules* **22**, 1207 (2017).

6. Ohyama, T. *et al.* Structure of Musashi1 in a complex with target RNA: the role of aromatic stacking interactions. *Nucleic Acids Res* **40**, 3218–3231 (2012).

7. Murn, J., Teplova, M., Zarnack, K., Shi, Y. & Patel, D. J. Recognition of distinct RNA motifs by the clustered CCCH zinc fingers of neuronal protein Unkempt. *Nat Struct Mol Biol* **23**, 16–23 (2016).

8. Uren, P. J. *et al.* RNA-binding protein Musashi1 is a central regulator of adhesion pathways in glioblastoma. *Mol Cell Biol* **35**, 2965–2978 (2015).

9. Murn, J. *et al.* Control of a neuronal morphology program by an RNA-binding zinc finger protein, Unkempt. *Genes Dev* **29**, 501–512 (2015).

10. Katz, Y. *et al.* Musashi proteins are post-transcriptional regulators of the epithelial-luminal cell state. *Elife* **3**, e03915 (2014).
