# Supplementary figures and images for: Dissecting RNA selectivity mediated by tandem RNA-binding domains
Source: J Biol Chem. 2025 Mar 20;301(5):108435. doi: 10.1016/j.jbc.2025.108435 (PMC12136788; doi:10.1016/j.jbc.2025.108435)

Supp. Figure 1

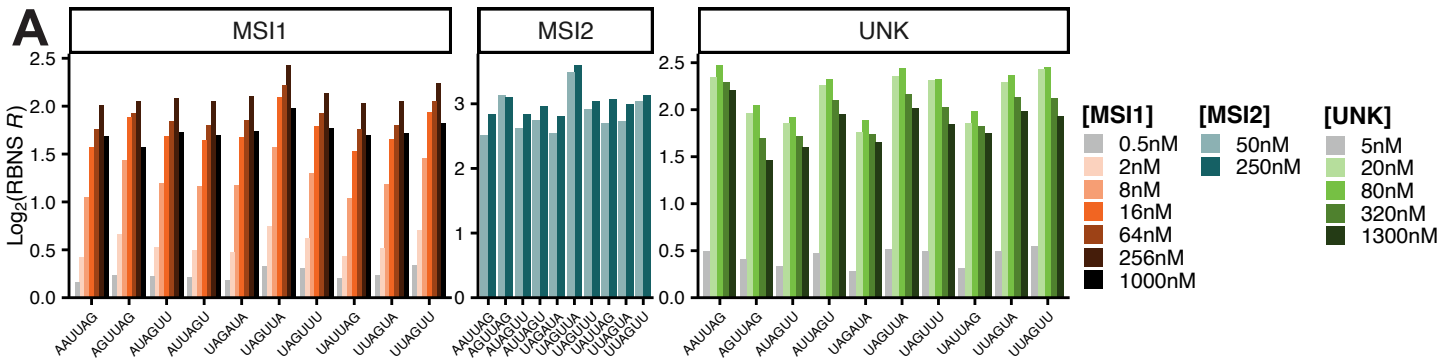

## Supp. Figure 2

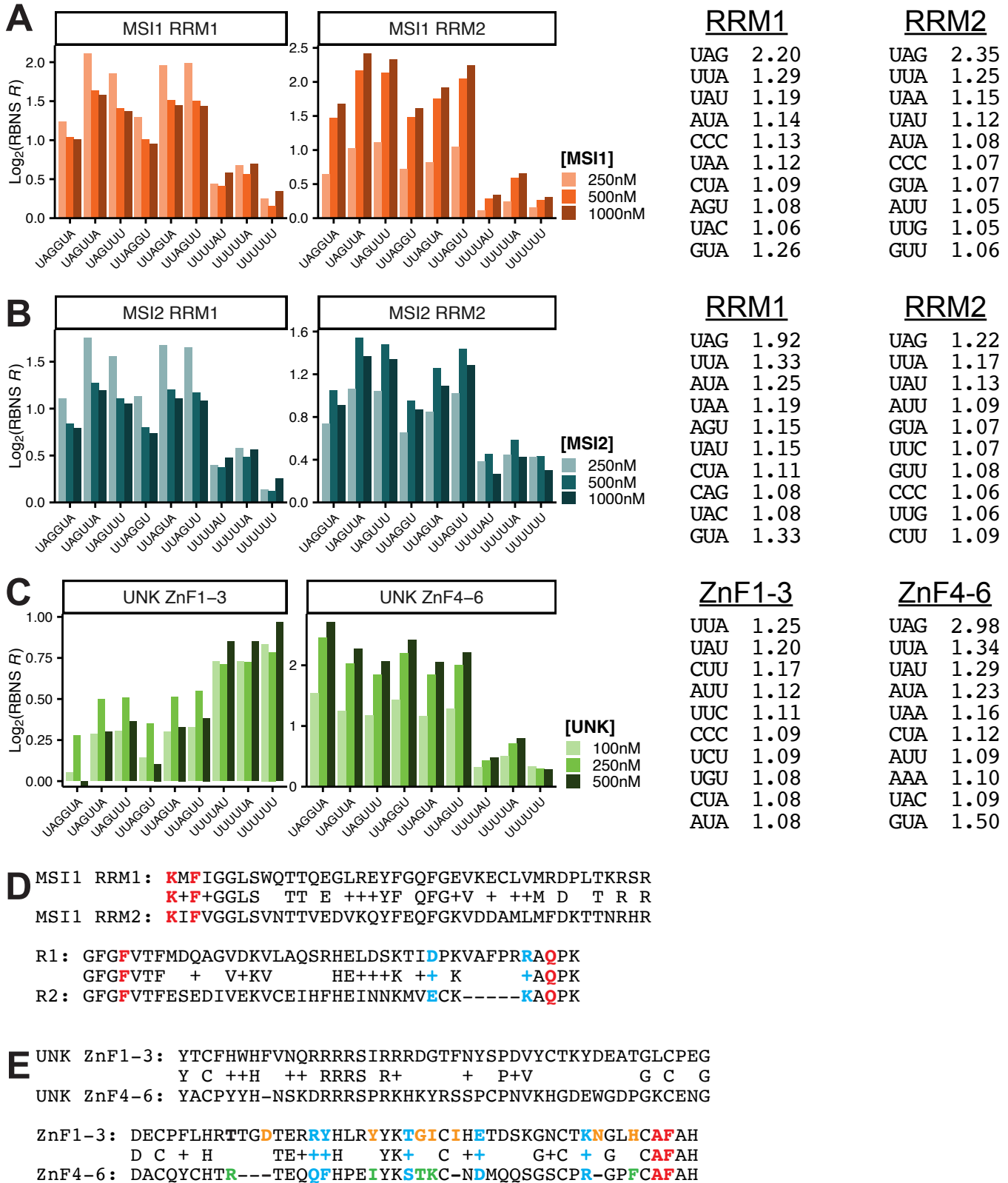

### Supp. Figure 3

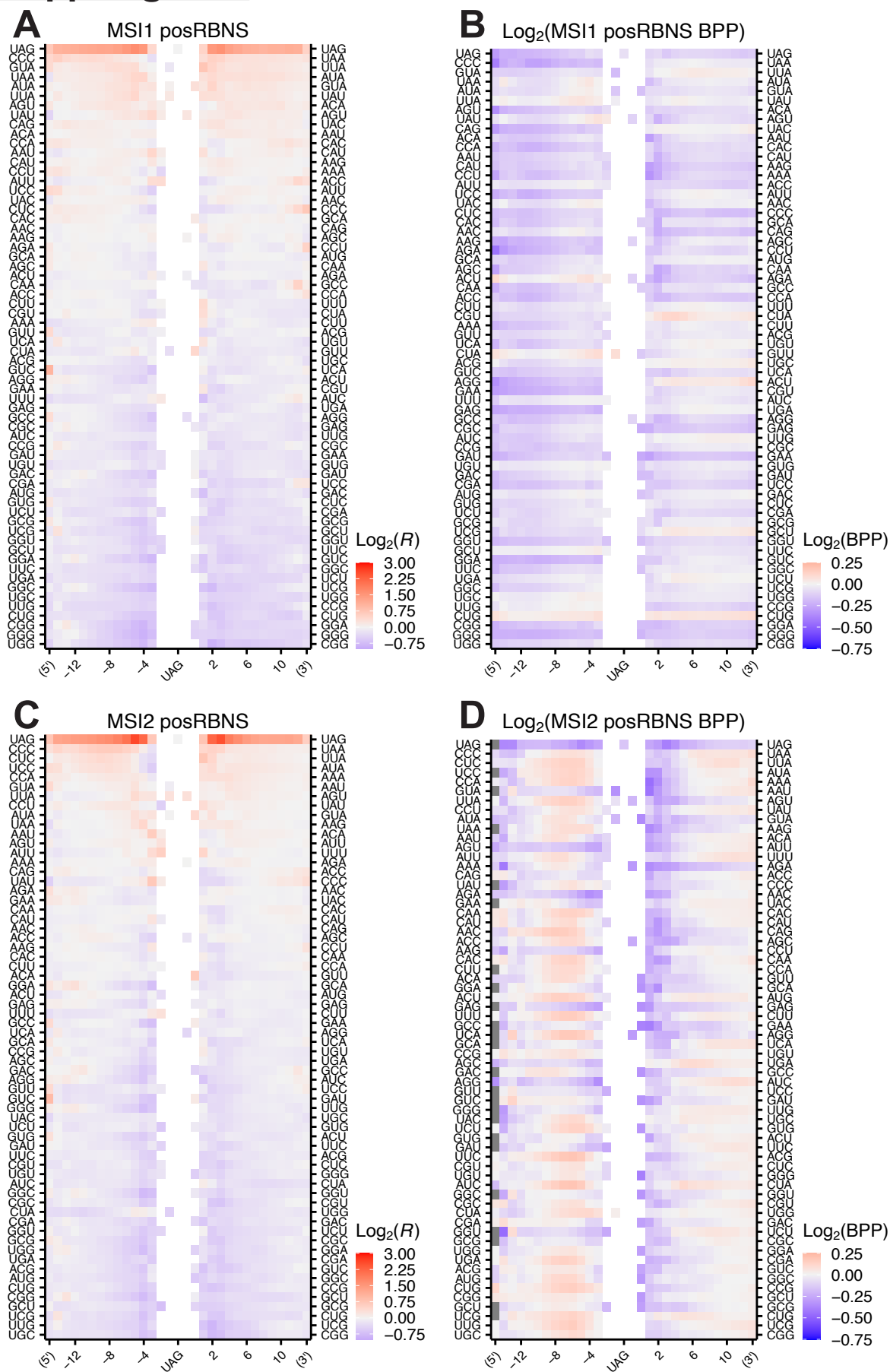

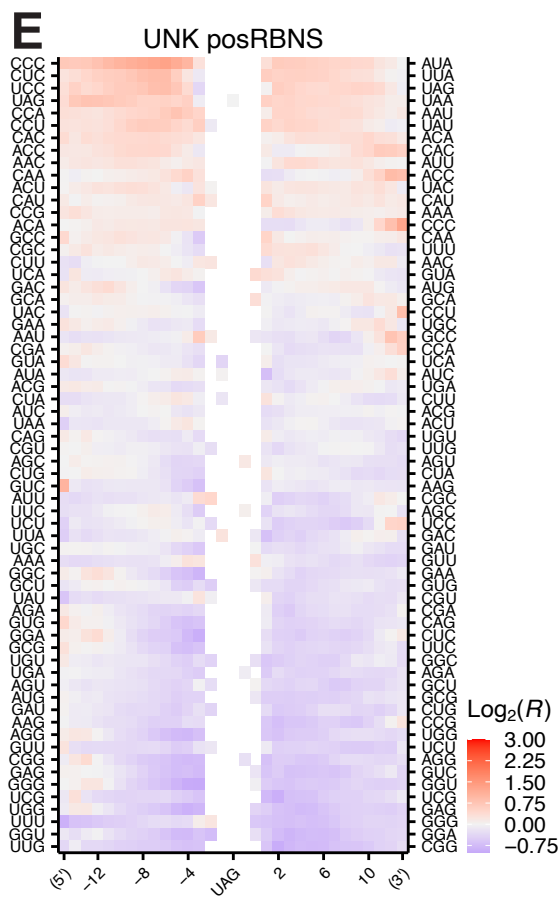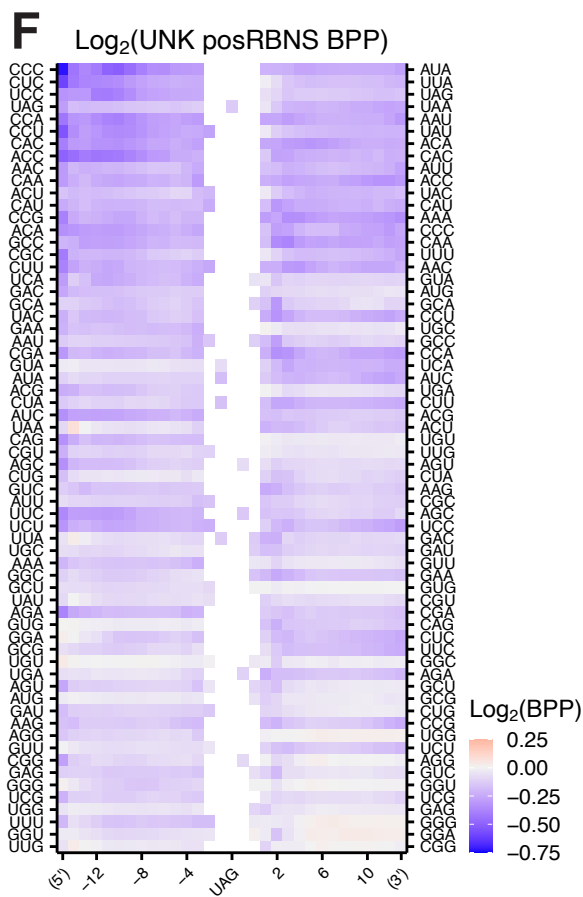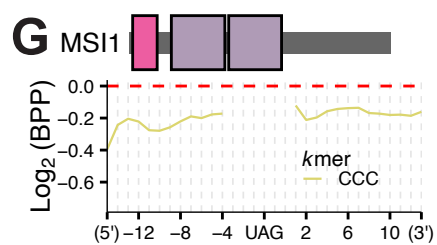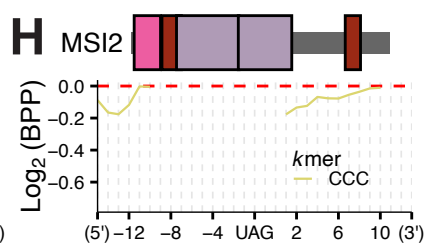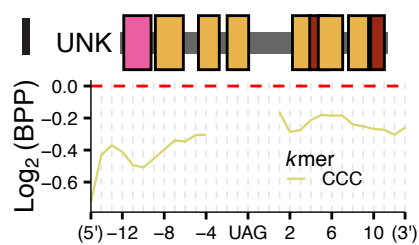

Supp. Figure 4

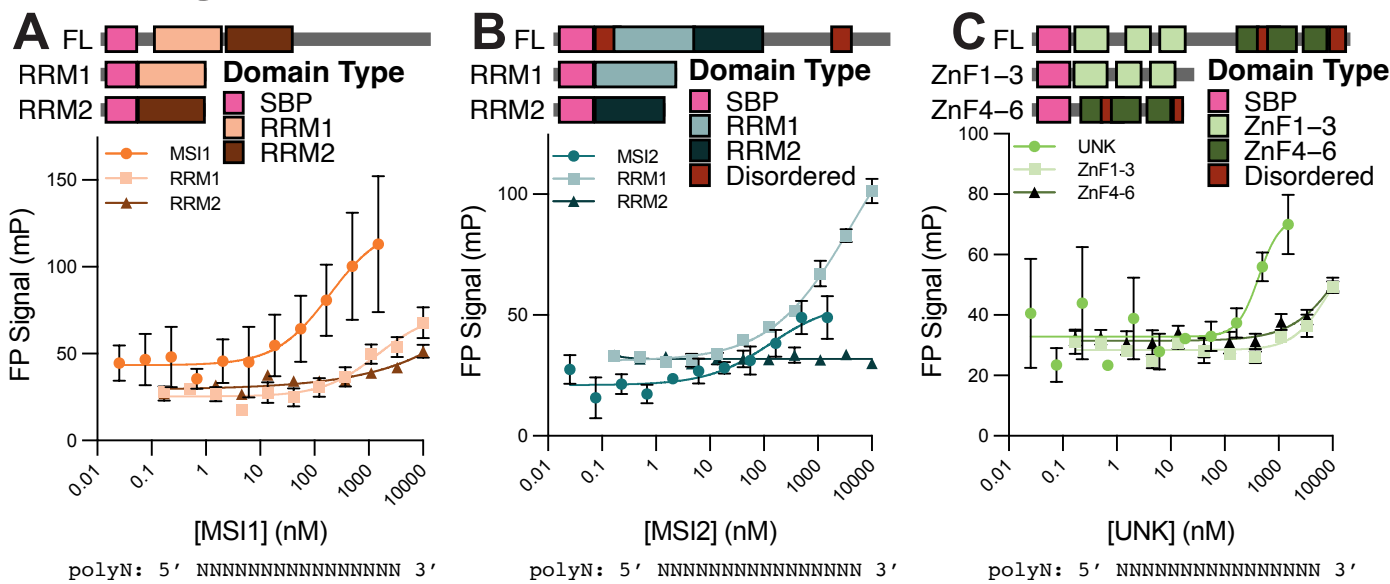

## Supp. Figure 5

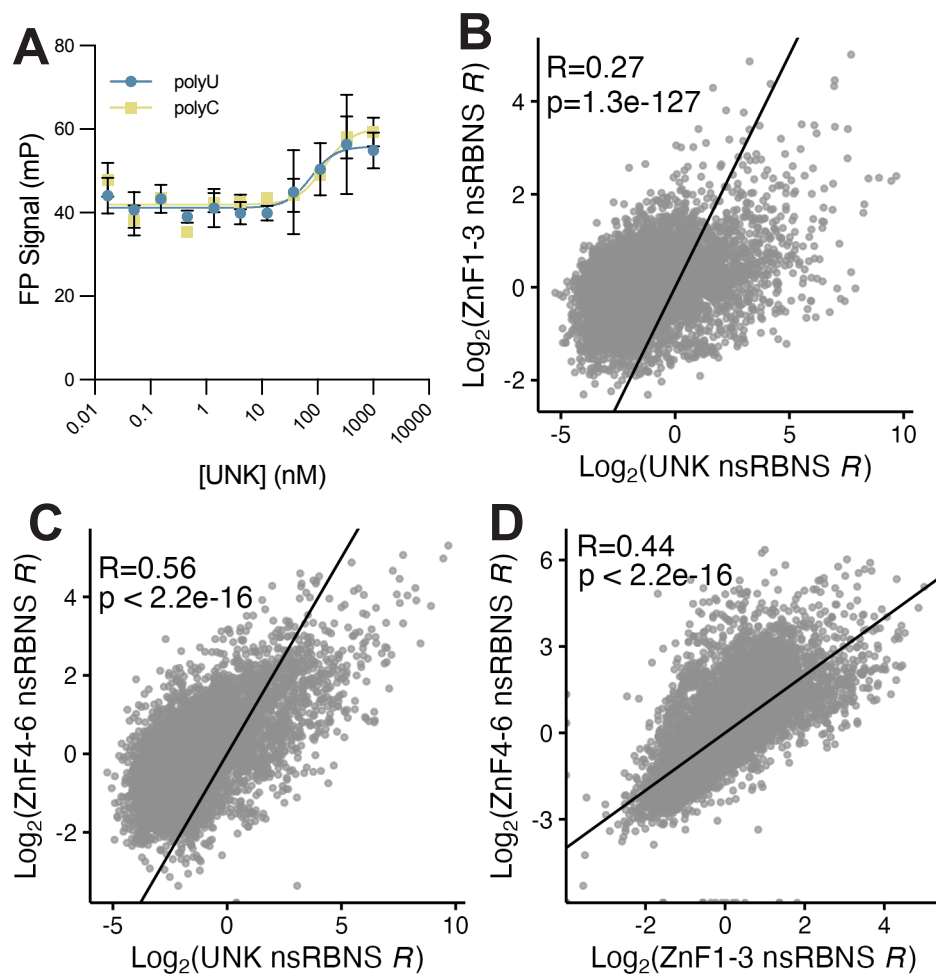

Supp. Figure 6

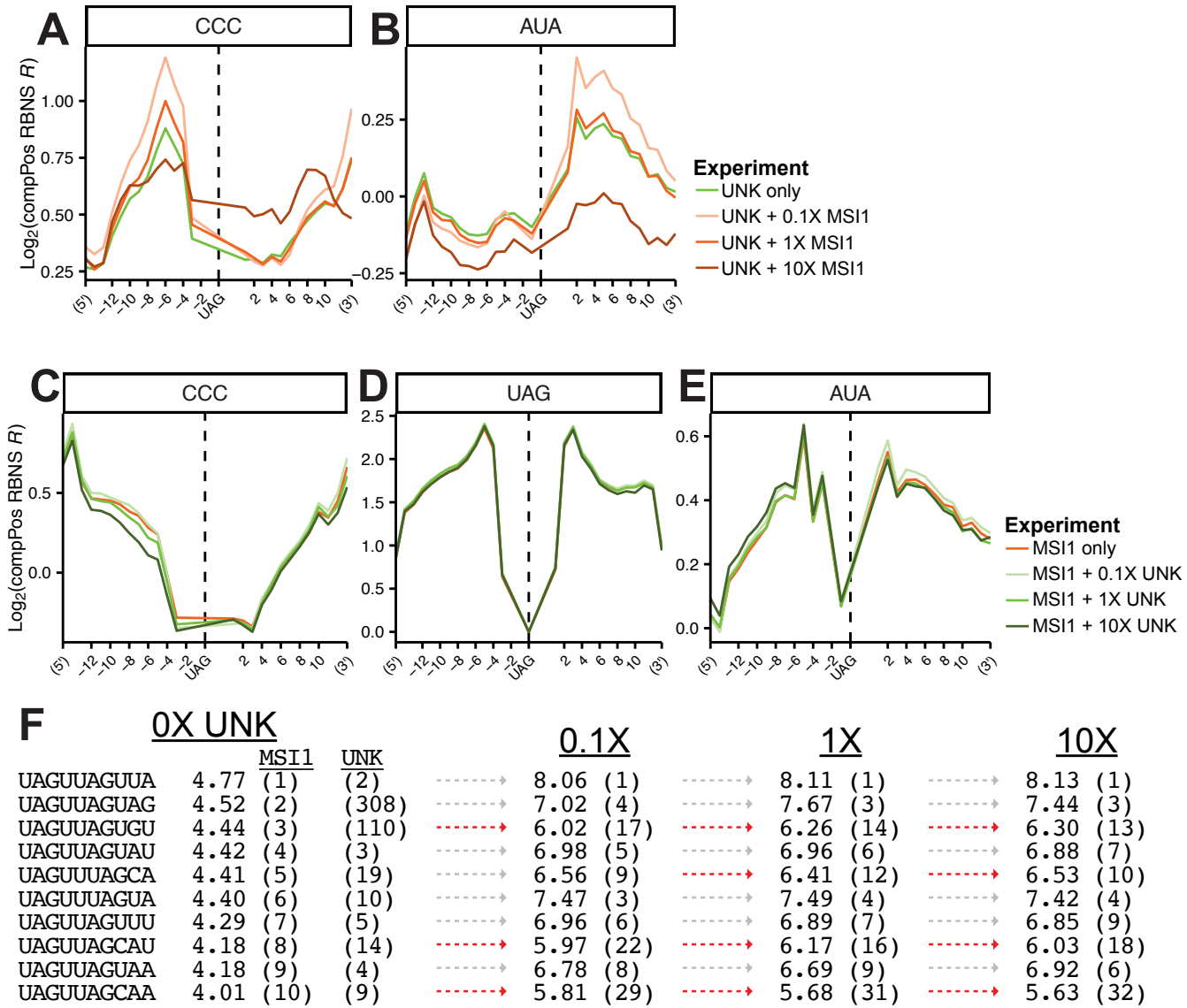

# Supp. Figure 7

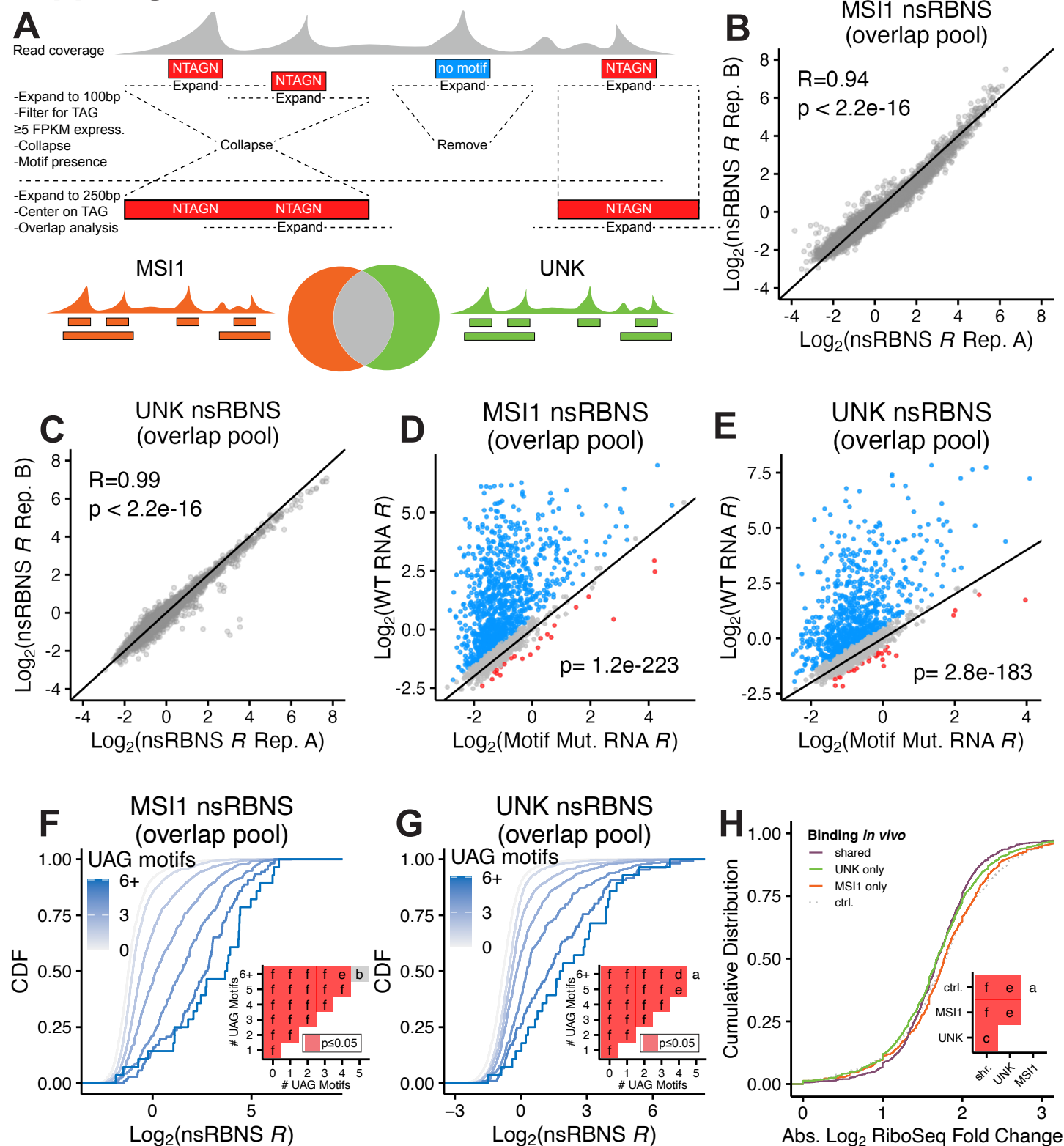

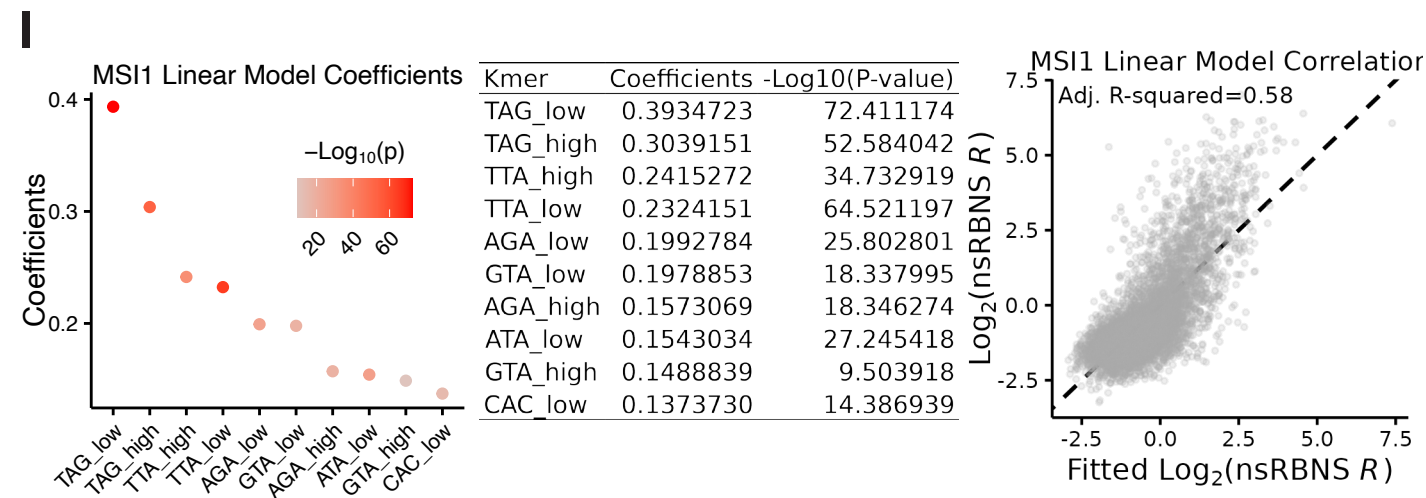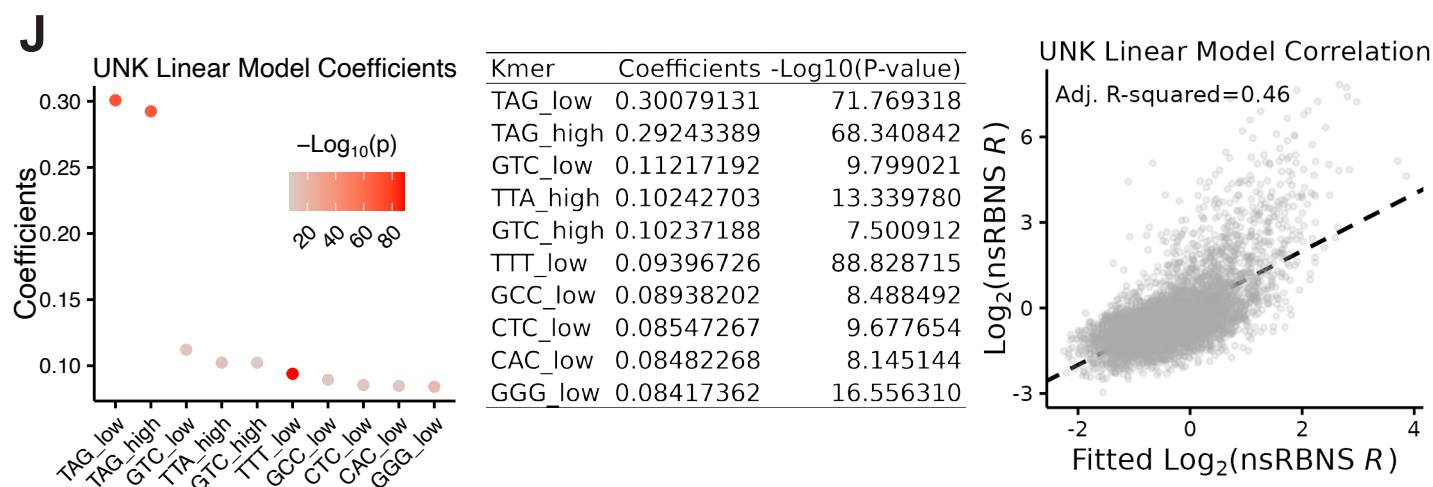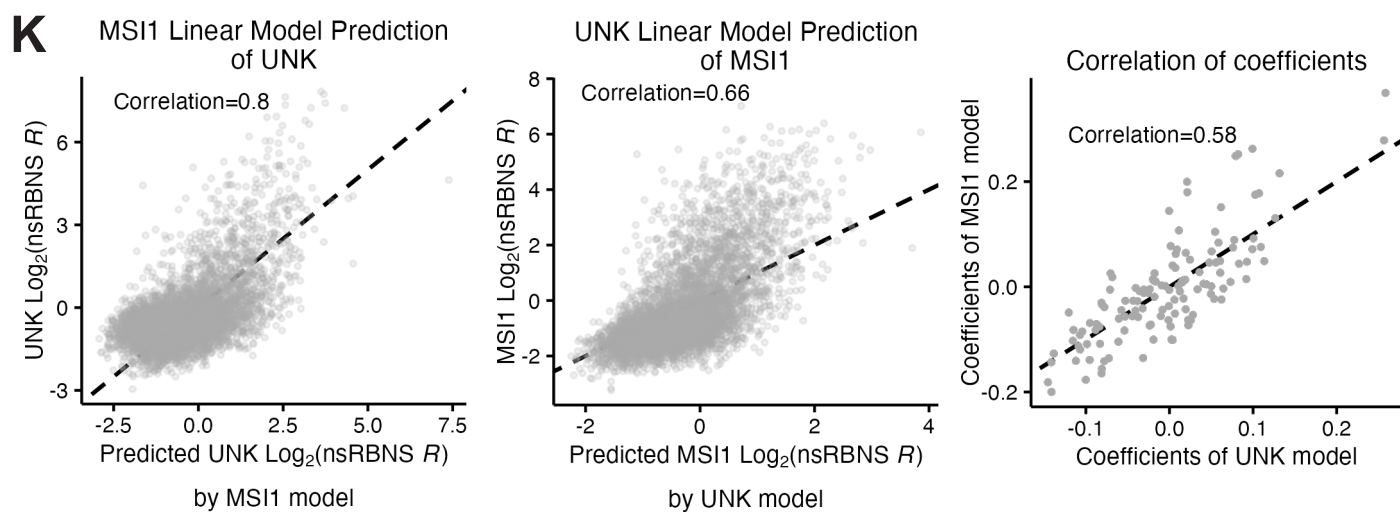

Supplement: Supp_figures_compiled [file mmc2.pdf]
